# Identifying mutation regions for closely related individuals without a known pedigree

**DOI:** 10.1186/1471-2105-13-146

**Published:** 2012-06-25

**Authors:** Wenjuan Cui, Lusheng Wang

**Affiliations:** 1 Department of Computer Science, City University of Hong Kong, Kowloon, Hong Kong

## Abstract

**Background:**

Linkage analysis is the first step in the search for a disease gene. Linkage studies have facilitated the identification of several hundred human genes that can harbor mutations leading to a disease phenotype. In this paper, we study a very important case, where the sampled individuals are closely related, but the pedigree is not given. This situation happens very often when the individuals share a common ancestor 6 or more generations ago. To our knowledge, no algorithm can give good results for this case.

**Results:**

To solve this problem, we first developed some heuristic algorithms for haplotype inference without any given pedigree. We propose a model using the parsimony principle that can be viewed as an extension of the model first proposed by Dan Gusfield. Our heuristic algorithm uses Clark’s inference rule to infer haplotype segments.

**Conclusions:**

We ran our program both on the simulated data and a set of real data from the phase II HapMap database. Experiments show that our program performs well. The recall value is from 90% to 99% in various cases. This implies that the program can report more than 90% of the true mutation regions. The value of precision varies from 29% to 90%. When the precision is 29%, the size of the reported regions is three times that of the true mutation region. This is still very useful for narrowing down the range of the disease gene location. Our program can complete the computation for all the tested cases, where there are about 110,000 SNPs on a chromosome, within 20 seconds.

## Background

Linkage analysis is the first step in the search for a disease gene. The aim is to find the rough location (a region in which the disease gene is) of the gene in the chromosome. Linkage studies have facilitated the identification of several hundred human genes that can harbor mutations leading to a disease phenotype [1]. The principle of linkage analysis is simple. All our chromosomes come in pairs, one inherited from the mother and the other from the father. Each pair of chromosomes contains the same genes in the same order, but the sequences are not identical. Thus it is possible to find out whether a particular sequence comes from the mother or father. These sequence variants are called maternal and paternal alleles. The key problem for linkage analysis is to infer the pairs of alleles and identify regions whose allele is shared by all or most of the diseased individuals but by none or few of the normal individuals.

Linkage analysis has been extensively studied in recent years. Almost all the existing methods are for families with clearly given pedigrees. The pedigree information helps a lot in the design of computational methods. Early approaches to linkage analysis were based on sparse microsatellite markers. With the new development of microarray techniques, high-density SNP genotype data can be used for large-scale and cost-effective linkage analysis [2,3]. With high-density SNP genotype data, there exists a sufficient number of informative markers between every pair of recombination points, and the allele-sharing status among the family members can be unambiguously determined. Lots of new computer programs have been developed for dealing with high-density SNP genotype data.

There are two categories of existing approaches to linkage analysis, the probabilistic approaches and the deterministic approaches. In probabilistic approaches, recombinant rates are estimated in a way to maximize the likelihood of the observed data [4-7]. Software tools based on this kind of approach include GeneHunter [5], LINKAGE [8], Allegro [6], Merlin [7], etc. According to [2], these tools have different performances and efficiencies. Some of them (such as those based on the Elston-Steward algorithm [9]) do not work well when the number of markers is large, while the others (such as those based on the Lander-Green algorithm [4]) do not work well with large number of family members. Though tremendous improvement has been made to them through subsequent modifications [6,7], this still remains a problem in practice. On the other hand, these tools can give very accurate results when the size of the pedigree is small.

Some deterministic approaches have been developed recently. The main idea is to infer the haplotype segments based on the input genotype data so that all or most of the diseased individuals share a segment that is shared by none of the normal individuals [10,11]. The mathematical model used here is to minimize the total number of recombinants among all the individuals in the pedigree. Lots of algorithms for haplotype inference with a pedigree have been developed. Qian and Beckmann [12] and Tapader *et al.*[13] proposed a method to minimize the number of recombinants with a given pedigree. Zhang *et al.*[14] developed a program for general pedigrees assuming that there is no recombinant on the segment. Doi *et al.*[15] designed two algorithms for haplotype inference with a given pedigree. One of their algorithms works well when the number of marker loci is a fixed constant, while the other works well when the number of family members is bounded by a small constant. Li and Jiang [16,17] proposed to use an integer linear programming approach for minimum recombinant configuration. Xiao *et al.*[18] designed a faster algorithm for the case where there is no recombinant. The algorithm in [10] uses a set of heuristics for haplotype inference with a given pedigree and can give very accurate results when the number of family members is large enough and for each nuclear family the genotype data for both parents are available. An extended software package (called LIden) was developed in [19] and it focuses on handling the case where the genotype data for the whole chromosome of one of the parents in a nuclear family are missing. It also uses the minimum recombinant model for haplotype inference in pedigrees.

Throughout this paper, we study the dominant inheritance situation, where sharing of one mutation allele can cause a disease phenotype. We deal with a very important case, where the sampled individuals are closely related, but the pedigree is not given. This situation happens very often in lots of villages in China when the individuals share a common ancestor 6 or more generations ago. Handling this case will be very helpful to identify some local genetic diseases in China. The situation also happens when studying wild animals, where the pedigree can not be identified. To our knowledge, no algorithm can give good results for this case. To solve this problem, we first developed some heuristic algorithms for haplotype inference without any given pedigree. We propose a model using the parsimony principle that can be viewed as an extension of the model first proposed in [20,21]. Our heuristic algorithm uses Clark’s inference rule [22] to infer haplotype segments. Experiments show that our program performs well. The recall value is from 90% to 99% in various cases. This implies that the program can report more than 90% of the true mutation regions. The value of precision varies from 29% to 90%. When the precision is 29%, the size of the reported regions is three times that of the true mutation region. This is still very useful for narrowing down the range of the disease gene location. Our program can complete the computation for all the tested cases, where there are about 110,000 SNPs on a chromosome, within 20 seconds.

## Implementation

Our software is implemented in Java. It takes the genotype data on a chromosome as well as the disease status for a set of input individuals without any pedigree information as input, and outputs the predicted mutation regions. The software is platform independent. In the following, we show the methods we used and how we implemented our algorithm.

### The problem

**Figure 1:**
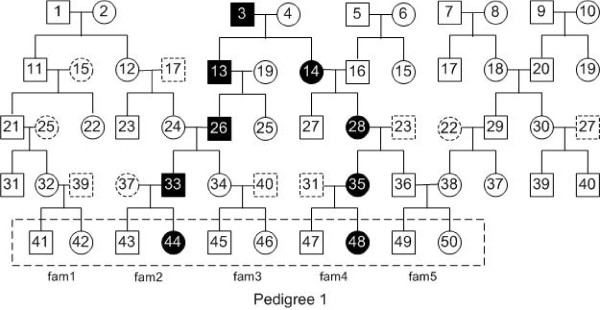
**Pedigree 1: a pedigree with 2 diseased individuals in the input.** There are 5 generations in the pedigree. The filled squares (circles) represent the diseased individuals. In the latest generation of this pedigree, 2 out of 10 individuals are diseased, which are numbered 44 and 48.

Suppose that there is a (hidden) pedigree containing many (e.g., 5 to 7) generations, where we only have the genotype data on a chromosome for the individuals in the latest generation (or the latest two generations). Here those individuals with given genotype data are referred to as the *input* individuals. For each input individual, we also know if such an individual is diseased or normal. An example is given in Figure 1, where there are five generations in the pedigree and we only have the genotype data for the individuals in the dashed rectangle at the bottom of the pedigree. In such a figure, a square represents a male, while a circle represents a female. Moreover, a filled square (respectively, circle) represents a diseased male (respectively, female), while an unfilled square (respectively, circle) represents a normal male (respectively, female). Furthermore, if two squares (respectively, circles) enclose the same number in the figure, then they correspond to the same male (respectively, female) and their sides (respectively, circumferences) are dashed.

For a genotype segment *g* of length *L*, the value at each position of *g* can be 0,1, or 2. A position of *g* with 0 indicates that both haplotypes have 0 at this position, while a position of *g* with 1 indicates that both haplotypes have 1 at this position. If the value at a position is 2, then one of the haplotypes is 0 while the other is 1 at this position. A pair of haplotype segments (*h*,*h*^*′*^) is a *haplotype pair* for a genotype segment *g* if they satisfy the following conditions:

C1. If the value of *g* is 0 (or 1) at a position, the values of *h* and *h*^*′*^ at this position are both 0 (or 1).

C2. If the value of *g* is 2 at a position, one of *h* and *h*^*′*^ is 0 and the other is 1 at this position.

We also say that the pair of haplotype segments (*h*,*h*^*′*^) can *explaing*.

Throughout this paper, we study the dominant inheritance situation, where sharing of one mutation allele can cause a disease phenotype. The general problem is as follows: we are given two sets of genotypes on the whole chromosome *D* = {*G*_1_,*G*_2_,…,*G*_*k*_} and *N* = {*G*_*k* + 1_,*G*_*k* + 2_,…,*G*_*n*_}, where the *k* genotypes in *D* are from diseased individuals and the *n* − *k* genotypes in *N* are from normal individuals. The *n* individuals in *D* and *N* are closely related (in the same hidden pedigree). The objective is to detect the *mutation regions* on the chromosome, where all the diseased individuals share a common haplotype segment on the mutation region and none of the normal individuals has such a common haplotype segment on the mutation region. Note that each individual has two haplotype segments on each region. If we know the haplotypes of each input individual over the chromosome, the *shared mutation regions* can be computed by finding the haplotype segments which are shared by all the diseased individuals but by none of the normal ones. The *true* mutation region is a shared mutation region containing the disease gene. Therefore, to solve the problem, the key issue is to infer the haplotype segments.

The task of inferring the haplotypes of each individual over the whole chromosome is extremely hard. For our purpose, we divide the whole chromosome into a set of disjointed length *L* segments, where *L* is a parameter to be determined later. For each length *L* segment, we try to infer the two haplotype segments of each individual based on the following mathematical model.

#### Mutation Region Haplotype Inference Problem (MRHIP)

Given two sets $D^{R}=\{g_{1}^{R},g_{2}^{R},\ldots ,g_{k}^{R}\}$ and $N^{R}=\{g_{k+1}^{R},g_{k+2}^{R},\ldots ,g_{n}^{R}\}$ of genotype segments on a length *L* region *R*, where the first *k* genotype segments in *D*^*R*^ are from diseased individuals and the *n* − *k* genotype segments in *N*^*R*^ are from normal individuals, we want to compute a center haplotype segment *h*^*R*^ and a pair of haplotype segments $h_{i,1}^{R}$ and $h_{i,2}^{R}$ for each $g_{i}^{R}$ in *D*^*R*^∪*N*^*R*^, such that the following conditions hold:

(1)$h_{i,1}^{R}=h^{R}$ for any $g_{i}^{R}\in D^{R}$.

(2)$h_{t,r}^{R}\neq h^{R}$ for $g_{t}^{R}\in N^{R}$ and *r* = 1,2;

(3)the total number *x*_*R*_ of distinct haplotype segments on *R* is minimized.

Without loss of generality, we assume that all the genotype segments on *R* in *D*^*R*^ are distinct. Similarly, all the genotype segments on *R* in *N*^*R*^ are also distinct. However, a genotype segment on *R* from a diseased individual and a genotype segment on *R* from a normal individual may be identical. In this case, such a genotype segment on *R* should be in both *D*^*R*^ and *N*^*R*^.

Condition (1) makes sure that all the diseased individuals have a haplotype segment which is identical to the center haplotype segment *h*^*R*^. Condition (2) ensures that all the normal individuals do not have *h*^*R*^. Condition (3) uses the parsimony principle, i.e., we want the total number of distinct haplotype segments to be minimized. Due to condition (3), this mathematical model can be viewed as an extension of the parsimony model first proposed by Gusfield in [20] for haplotype inference. The parsimony model has been extensively studied in [20,21,23,24]. MRHIP can be viewed as a simplified version of the Maximum Resolution (MR) Problem which is proved to be NP-hard in [20].

It should be emphasized that for some input of MRHIP on a region *R*, the solution of MRHIP may not exist. Even if the solutions exist, the values of *x*_*R*_ may vary for different inputs. If *R* is the mutation region, the solution for MRHIP on *R* always exists and the value of *x*_*R*_ should be small.

Our approach contains three steps. First, we decompose the whole chromosome into a set of disjointed length *L* (*L* = 500) segments and try to give a solution for MRHIP on each length *L* segment. We then have an algorithm to merge length *L* segments based on the computational results to form longer segments and try to get solutions for MRHIP on those longer segments. After that, we have a method to further extend the longer segments to the left and right. Finally, our algorithm reports all the detected mutation regions.

### The algorithm for MRHIP

For the Mutation Region Haplotype Inference Problem(MRHIP), we designed an algorithm to solve it. Given an instance of MRHIP, there may or may not exist a solution. If a solution does not exist, there are two cases:

1.There does not exist a center haplotype *h*^*R*^ which is shared by all the genotype segments in *D*^*R*^. This case is referred to as type I. Type I cases occur when one element in *D*^*R*^ has genotype value 0 and the other element in *D*^*R*^ has genotype value 1.

2.We can find a center haplotype *h*^*R*^, but some genotype segments in *N*^*R*^ must be explained by a pair of haplotype segments and one of the haplotype segments is identical to *h*^*R*^. This case is referred to as type II.

An example of a type II case is the following: *D*^*R*^ = {*g*_1_ = 111,*g*_2_ = 121}, and *N*^*R*^ = {*g*_3_ = 112,*g*_4_ = 102}. Based on *g*_1_ and *g*_2_, the shared center haplotype *h*^*R*^ must be 111. However, *g*_3_ indicates that normal individuals also have a haplotype segment 111 on *R* which is identical to the shared center *h*^*R*^. Thus, condition (2) in MRHIP does not hold.

In our algorithm, we first compute the center haplotype *h*^*R*^ based on the diseased genotype segments in *D*^*R*^. We look at the positions in *R* one by one. Based on C1 and C2, if one of the diseased individuals has genotype value 0, then the haplotype value of *h*^*R*^ at this position should be 0; if one of the diseased individuals has genotype value 1 at a position, then the haplotype value of *h*^*R*^ at this position should be 1. If there exists a position *p* at which one diseased individual has genotype value 0 and the other diseased individual has genotype value 1, then a *conflict* occurs and position *p* is called a *conflicting position*. Once a conflict occurs, we simply conclude that there is no MRHIP solution on this segment *R*. We say the Type I False occurs in this case. If all the diseased individuals have genotype value 2 at a position *p* in *R*, then the haplotype value of *h*^*R*^ cannot be determined at this step. We call such a position the *wild card* position and put a ∗ at the wild card position to indicate that the haplotype value of *h*^*R*^ will be determined later. The detailed procedure (Procedure P1) for computing the center haplotype segment *h*^*R*^ is given as follows:

**for** each position *p* in *R***do**

1.if all the diseased individuals have genotype value 0 or 2, then set the haplotype value of *h*^*R*^ at *p* to 0.

2.if all the diseased individuals have genotype value 1 or 2, then set the haplotype value of *h*^*R*^ at *p* to 1.

3.if some diseased individuals have genotype value 0 and some other diseased individuals have genotype value 1, then return Type I False.

4.if all the diseased individuals have genotype value 2, then set the haplotype value of *h*^*R*^ to ∗ (indicating that the haplotype value of *h*^*R*^ will be determined later).

Without loss of generality, for each diseased individual $g_{i}^{R}\in D^{R}$, we set $h_{i,1}^{R}=h^{R}$ for *i* ≤ *k*. Then we can set $h_{i,2}^{R}$ in such a way that $\left(h_{i,1}^{R},h_{i,2}^{R}\right)$ is a haplotype pair for $g_{i}^{R}$ with *i* ≤ *k*. Note that, the values of $\left(h_{i,1}^{R},h_{i,2}^{R}\right)$ at wild card positions in *R* are still not yet determined. Here we refer to $h_{i,2}^{R}$ as a *partially* inferred haplotype segment on *R*. Let $\mathrm{intQ}=\{h_{1,2}^{R},h_{2,2}^{R},\ldots ,h_{k,2}^{R}\}$, where if two haplotype segments $h_{i,2}^{R}$ and $h_{i^{\prime },2}^{R}$ are identical, then we just keep one of them. Note that if any haplotype segment $h_{i,2}^{R}\in \mathrm{intQ}$ is undetermined at a position *p*, then all the haplotype segments in *intQ* are undetermined at *p*.

After partially determining *h*^*R*^ and $\left(h_{i,1}^{R},h_{i,2}^{R}\right)$ for every $g_{i}^{R}$ in *D*^*R*^, we use a heuristic method to infer the haplotype segments for $g_{i}^{R}\in N^{R}$. Since we want to minimize the total number of resulting distinct haplotype segments on *R*, our strategy is to let the inferred haplotype segments for $g_{i}^{R}\in N^{R}$ share as many haplotype segments as possible. This is actually Clark’s inference rule [22].

Let *Q* be a queue that contains a set of (partially) inferred haplotype segments on *R*. Initially, *Q* = *intQ*. A partially inferred haplotype segment *h* in *Q* can *solve*$g_{j}^{R}$ if the following conditions hold:

1.if *h* is 0 at a position *p* then $g_{j}^{R}$ is 0 or 2 at position *p*.

2.if *h* is 1 at a position *p* then $g_{j}^{R}$ is 1 or 2 at position *p*.

We can use *h* to *solve*$g_{j}^{R}$ by constructing two haplotype segments $\left(h_{j,1}^{R},h_{j,2}^{R}\right)$ as follows:

Using *h* to solve $g_{j}^{R}$:

1.if *h* is 0 at position *p* in *R* then we set $h_{j,1}^{R}=0$ at *p* and $h_{j,2}^{R}$ at *p* is set according to rules C1 and C2.

2.if *h* is 1 at position *p* in *R* then we set $h_{j,1}^{R}=1$ at *p* and $h_{j,2}^{R}$ at *p* is set according to rules C1 and C2.

3.if *h* is undetermined at position *p* in *R*, and $g_{j}^{R}$ is 0 (or 1) at *p*, then set $h_{j,1}^{R}=h=h_{j,2}^{R}=0$ (or $h_{j,1}^{R}=h=h_{j,2}^{R}=1$) at *p*. Here we also have to determine the value of *h* at position *p* accordingly. After the undetermined value of *h* at *p* is determined, if *h* is obtained from a $g_{i}^{R}\in D^{R}$, then we also have to determine the values of *h*^*R*^ and other $h_{i,2}^{R}$ for $g_{i}^{R}\in D^{R}$ at position *p* according to the haplotype value of *h* at *p* and rules C1 and C2.

4.if *h* is undetermined at position *p* in *R*, and $g_{j}^{R}$ is 2 at *p*, then $h_{j,1}^{R}$ and $h_{j,2}^{R}$ remain undetermined at *p*.

A genotype segment $g_{i}^{R}\in N^{R}$ is *solved* if the pair of haplotype segments $\left(h_{i,1}^{R},h_{i,2}^{R}\right)$ for $g_{i}^{R}$ are (partially) determined. In our algorithm, we use *P* to store the set of genotypes in *N*^*R*^ that have not been solved. Initially, *P* = *N*^*R*^. We then use the haplotype segments in *Q* one by one and try to solve each of the genotypes in *P*. After trying to use a *h* ∈ *Q* to solve all $g_{j}^{R}$’s in *P*, we delete *h* from *Q*. Two haplotype segments on *R* in *Q* are *compatible* if there does not exist any position *p* in *R* such that one segment has value 0 and the other segment has value 1 at *p*. For two compatible haplotype segments *h*_1_ and *h*_2_ on *R*, we can *merge* them to form one haplotype segment *h*, where the value of *h* is determined as 0 or 1 if at least one of *h*_1_ and *h*_2_ is 0 or 1 and the value of *h* remains undetermined if both *h*_1_ and *h*_2_ are undetermined. Again, for any position *p*, if the value of *h*_1_ or *h*_2_ is not identical to that of *h*, then the value of *h*_1_ or *h*_2_ is changed from undetermined to 0 or 1. Thus, we have to update some of the previously inferred haplotype segments accordingly.

When we use a *h* ∈ *Q* to solve a $g_{j}^{R}$ in *P*, we can obtain another new haplotype segment *h*^*′*^ on *R*. If *h*^*′*^ is compatible with a haplotype segment in *Q*, we then merge them. Note that, *h*^*′*^ might be compatible with more than one haplotype segment in *Q*. In this case, we arbitrarily choose a compatible haplotype segment in *Q* and merge the two haplotype segments. If *h*^*′*^ is not compatible with any haplotype segment in *Q*, we add *h*^*′*^ into *Q*.

We give an example to illustrate the above process.

#### Example 1

*D*^*R*^ = {*g*_1_ = 10211,*g*_2_ = 12221,*g*_3_ = 10221} and *N*^*R*^ = {*g*_4_=10121}. After Procedure P1, *h*^*R*^ = 10∗11 and consequently *intQ* = {*h*_1,2_ = 10∗11,*h*_2,2_ = 11∗01,*h*_3,2_ = 10∗01}. After that , we can use *h*_1,2_ = 10∗11 in *intQ* to solve *g*_4_ = 10121 in *N*^*R*^. Based on *h*_1,2_ = 10∗11, *g*_4_ = 10121 can be solved as *h*_4,1_ = 10111 and *h*_4,2_ = 10101. Moreover, since we want *h*_1,2_ and *h*_4,1_ to be identical (to minimize the number of distinct haplotype segments), *h*_1,2_ is updated as *h*_1,2_ = 10111. Correspondingly, we update *h*^*R*^ = *h*_1,1_ = 10011, *h*_1,2_ = 10111, *h*_2,2_ = 11101,and *h*_3,2_ = 10101. After that, the set of distinct haplotype segments we have obtained so far is {*h*^*R*^ = 10011,*h*_1,2_ = *h*_4,1_ = 10111,*h*_2,2_ = 11101,*h*_3,2_ = 10101,*h*_4,2_ = 10101}. Note that *h*_3,2_ and *h*_4,2_ are compatible (actually identical), and the set of distinct haplotype segments is {*h*^*R*^ = 10011,*h*_1,2_ = *h*_4,1_ = 10111,*h*_2,2_ = 11101,*h*_3,2_ = *h*_4,2_ = 10101}.

After trying to use all the *h*’s in *Q* to solve all $g_{j}^{R}$’s in *P*, *Q* will become empty. When *Q* is empty and *P* still contains at least two genotype segments, we consider all pairs of genotype segments $g_{j}^{R}$ and $g_{j^{\prime }}^{R}$ in *P* and use the following method to infer the haplotype segments. Inferring the haplotype segments from a pair $g_{j}^{R}$ and $g_{j^{\prime }}^{R}$ in *P*:

A position *p* in *R* is a *conflicting* position for $g_{j}^{R}$ and $g_{j^{\prime }}^{R}$ if one of $g_{j}^{R}$ and $g_{j^{\prime }}^{R}$ has the genotype value 0 and the other has genotype value 1 at *p*. The pair of $g_{j}^{R}$ and $g_{j^{\prime }}^{R}$ can *share* a common haplotype segment on *R* if there is no conflicting position in *R* for $g_{j}^{R}$ and $g_{j^{\prime }}^{R}$. The shared haplotype segment can be computed as follows: (1) if one of the genotype values at position *p* is 0, then the haplotype value is 0 at *p*; (2) if one of the genotype values is 1 at *p*, then the haplotype value is 1 at *p*; (3 )if both genotype values are 2 at *p*, then the haplotype value at *p* is undetermined. Once the shared haplotype segment for $g_{j}^{R}$ and $g_{j^{\prime }}^{R}$ are computed, we can determine the other haplotype segments for $g_{j}^{R}$ and $g_{j^{\prime }}^{R}$ based on C1 and C2.

After inferring the haplotype segments from a pair $g_{j}^{R}$ and $g_{j^{\prime }}^{R}$ that can share a common haplotype segment, we delete $g_{j}^{R}$ and $g_{j^{\prime }}^{R}$ from *P*, merge compatible inferred haplotype segments, and insert the newly obtained haplotype segments into *Q*. Once *Q* is not empty, we can use haplotype segments in *Q* to solve the genotype segments in *P* again. The process is repeated until *P* is empty. The detailed algorithm is given as Algorithm 1.

### Algorithm 1

Mutation Region Haplotype Inference

**Input:** Two sets of genotype segments $D^{R}=\{g_{1}^{R},g_{2}^{R},\ldots ,g_{k}^{R}\}$ and $N^{R}=\{g_{k+1}^{R},g_{k+2}^{R},\ldots ,g_{n}^{R}\}$ on *R*.

**Output:True** if there is a solution. **Type I false** if two diseased haplotype segments are conflict at a position in *R*; **Type II false** otherwise.

1: Compute the center haplotype *h*^*R*^ as in Procedure P1.

2: **if***h*^*R*^ does not exist **then**

3: **return Type I False**

4: **else**

5:

set $h_{i,1}^{R}=h^{R}$ for $g_{i}^{R}\in D^{R}$;

6: compute $h_{i,2}^{R}$ according to $h_{i,1}^{R}$ and C1 and C2 for each $g_{i}^{R}\in D^{R}$; Set $Q=\{h_{1,2}^{R},h_{2,2}^{R},\ldots ,h_{k,2}^{R}\}$ (removing identical segments) and *P* = *N*^*R*^.

7: **end if**

8: **while***Q* ≠ *∅* and *P* ≠ *∅***do**

9: Delete a haplotype segment $h_{i,2}^{R}$ from *Q*;

10: **if**$h_{i,2}^{R}$ can solve $g_{j}^{R}$**then**

11: use $h_{i,2}^{R}$ to solve $g_{j}^{R}$. Add the newly obtained haplotype segments (after merging compatible segments) into *Q* and delete $g_{j}^{R}$ from *P*.

12: **end if**

13: **end while**

14: **if** there are at least 2 genotype segments in *P***then**

15: **if** there exists a pair of genotype segments $g_{j}^{R}$ and $g_{j^{\prime }}^{R}$ that can share a haplotype segment on *R***then**

16:

infer the haplotype segments of $g_{j}^{R}$ and $g_{j^{\prime }}^{R}$ and insert them (after merging) into *Q*, **goto** line 8.

17: **end if**

18: **if** any inferred haplotype segments for some $g_{j}^{R}\in N^{R}$ on *R* is identical to *h*^*R*^**then**

19: **return Type II False**.

20: **end if**

21:

fix $h_{s,1}^{R}$ and $h_{s,2}^{R}$ for each $g_{s}^{R}\in P$ so that $h_{s,1}^{R}\neq h^{R}$ and $h_{s,2}^{R}\neq h^{R}$.

22: **if** Line line 21 fails **then**

23: **return Type II False**

24: **else**

25: **return True**.

26: **end if**

27: **end if**

The following is an example to illustrate the case when *Q* becomes empty.

#### Example 2

*D*^*R*^ = {*g*_1_ = 12110,*g*_2_ = 11210} and *N*^*R*^ = {*g*_3_ = 21111,*g*_4_ = 11121}. After Procedure P1, *h*^*R*^ = 11110 and *intQ* = {10110,11010}. After trying all the *h*’s in *intQ* to solve *g*_*i*_’s in *N*^*R*^, *Q* becomes empty and *P* = *N*^*R*^  ={21111,11121}. In this case, we look at both 21111 and 11121 in *P* and infer a shared haplotype *h*_3,1_ = *h*_4,1_ = 11111 and the other two haplotype segments *h*_3,2_ = 01111 and *h*_4,2_ = 11101.

### The algorithm for the whole chromosome

For an input segment *R* on a chromosome, if Algorithm 1 returns true, then *R* is a *valid* segment. In order to get the mutation regions, we decompose the whole chromosome into a set of disjointed length *L* segments. (In this paper, we performed experiments on chromosomes with about 110,000 SNP sites. In this case, we set *L* = 500.) For each segment, we run Algorithm 1 to test if the segment is valid. After finding all the valid segments, we repeatedly merge two valid segments into a *long valid* segment if the two segments are within 2*L* SNPs and Algorithm 1 returns Type II false on all the segments in the gap.After the above merging process, we obtain several long valid segments. For each such long valid segment [*sb*,*se*), we run Algorithm 1 on the three segments [*sb*−0.5*L*,*se* + 0.5*L*), [*sb*−0.2*L*,*se* + 0.2*L*) and [*sb*,*se*) and select the longest one (denoted as [*b*,*e*)) which returns true. Since we impose that Algorithm 1 returns Type II false for the segments in gaps in the merging process, we can always ensure that Algorithm 1 returns true for [*sb*,*se*). Extending [*b,e*) to the left and right:

After we obtain *R* = [*b*,*e*) as discussed above, we try to extend the segment [*b*,*e*) to the left and right. On the segment *R*=[*b*,*e*), we have inferred $h_{i,1}^{R}$ and $h_{i,2}^{R}$ for each $g_{i}^{R}\in D^{R}\cup N^{R}$. These $h_{i,1}^{R}$ and $h_{i,2}^{R}$ form a collection of disjointed sets *H*_1_,*H*_2_,…,*H*_*m*_, where each *H*_*k*_(1 ≤ *k* ≤ *m*) is a set of identical haplotypes in $\left\{h_{i,1}^{R},h_{i,2}^{R}|i=1,2,\ldots ,n\right\}$ on *R*.

We extend the segment [*b*,*e*) to the left and right by looking at each position *p*. We first try *p* = *b* − *q* for *q* = 1,2,… (to the left) and then *p* = *e* − 1 + *q* for *q* = 1,2,… (to the right). For each *H*_*k*_(1 ≤ *k* ≤ *m*),

1.If there exist some *h*_*i*,*j*_ in *H*_*k*_ such that *g*_*i*_ are 0 (or 1) and others are 2, then every *h*_*i*,*j*_ in *H*_*k*_ should be 0 (or 1). If there exists a *h*_*i*,*j*_ in *H*_*k*_ that has been set to the conflict value 1 (or 0) before, then we know that position *p* is a conflicting position and *p* should not be extended to be part of *R* and the extension process to the current direction (left or right) should stop. Otherwise, we set every *h*_*i*,*j*_ in *H*_*k*_ to be 0 (or 1). If *p* is not a conflicting position, after setting *h*_*i*,*j*_ in *H*_*k*_ to be 0 (or 1), we can determine the value of $h_{i,j^{\prime }}$ (*j*^*′*^ = 1 if *j* = 2 and *j*^*′*^ = 2 if *j* = 1) according to the value of *h*_*i*,*j*_ at *p*, the value of *g*_*i*_ at *p* and rules *C*_1_ and *C*2. Again, we should test if such a value of $h_{i,j^{\prime }}$ is consistent with the value of $h_{i,j^{\prime }}$ determined before (if any). If conflict exists, then *p* is a conflicting position and the extension process to the current direction (left or right) should stop. Let $h_{i,j^{\prime }}\in H_{k^{\prime }}$. If there is no conflict, we should also update the value of all *h*’s in $H_{k^{\prime }}$. This recursive process continues until no further change can be made.

2.If all *h*_*i*,*j*_ in *H*_*k*_ are 2, we set *h*_*i*,*j*_ as undetermined.

The extension process stops when we find conflicts in both directions. The extended region obtained from [*b*,*e*) is denoted as [*rb*,*re*]. After the extension process, our program reports all the mutation regions obtained in the algorithm. The complete algorithm to find the mutation regions on the whole chromosome is shown as Algorithm 2.

### Algorithm 2

The algorithm for the whole chromosome

**Input:** Two sets of genotype on the whole chromosome *D* = {*G*_1_,*G*_2_,…,*G*_*k*_} and *N* = {*G*_*k* + 1_,*G*_*k* + 2_,…,*G*_*n*_}.

**Output:** The detected mutation regions

1:Decompose the whole chromosome into segments of length L = 500.

2:**for** each segment **do**

3:

use Algorithm 1 to test if the segment is valid.

4:**end for**

5:Merge the valid segments (see The algorithm for the whole chromosome in Implementation, the first paragraph) to form longer segments.

6:**for** each segment [*sb*,*se*) obtained in line 5 **do**

7:

Select the longest segment of [*sb* − 0.5*L*,*se* + 0.5*L*), [*sb* − 0.2*L*,*se* + 0.2*L*) and [*sb*,*se*) which will return true by calling Algorithm 1. Denote it as [*b*,*e*).

8:

Extend [*b*,*e*) to get a candidate mutation region.

9:**end for**

10:Output all the mutation regions obtained in the for loop of lines 6-9.

## Results

In this section, we first show some experiments on simulated data. We then give a real case study to show that our program can also handle real data (with errors). A discussion is given at the end of this section.

### Experiments on simulated data

In order to evaluate the performance of our method and the feasibility of the mathematical model proposed in this paper, we write a program in C++ to produce simulated data. The program takes a pedigree (e.g., Figure 1) and the haplotype data for the whole chromosome of each founder in the pedigree as the input. It generates the haplotype data for the remaining individuals in the pedigree using the standard *χ*^2^ model for recombination with *m* (the degree of freedom divided by 2) equal to 4 ([25]) and according to the male/female averaged genetic map for chromosome 1 downloaded from HapMap (http://hapmap.org). Also see [26]. The haplotype data of a non-founder in the pedigree are generated to randomly inherit one strand of the four-strand chromatid bundle from each parent of the non-founder. A mutation point is selected uniformly at random from the SNP sites of the chromosome. Each diseased offspring is forced to inherit (from each of its parents) the strand with the mutation point and the normal offspring are forced to inherit the strand without the mutation point. In this way, we can guarantee that there is exactly one *true mutation region*. Note that the true mutation region must be a shared mutation region. See Implementation for the definition. Moreover, since we know the haplotype data of all the individuals in the simulations, we can easily find the shared mutation regions. By definition, there may exist more than one shared mutation region.

To generate the simulated data, we randomly chose some of the haplotype data for chromosome 1 of 170 unrelated Japanese in Tokyo and Han Chinese in Beijing in the database of HapMap project (http://hapmap.ncbi.nlm.nih.gov/) as the haplotype data for each founder. There are about 110,000 SNP sites on this chromosome.

Recall that our program takes two sets of individuals *D* and *N* and their genotype data as input. After generating the haplotype data of each individual, we only use some of the individuals (in the dashed rectangle at the bottom of the pedigree, say, e.g., Figure 1) and their associated genotype (obtained from the simulated haplotype data) data as the input of our program.

**Figure 2:**
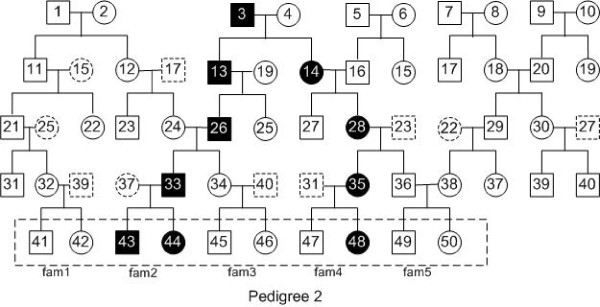
**Pedigree 2: a pedigree with 3 diseased individuals in the input.** There are 5 generations in the pedigree. The filled squares (circles) represent the diseased individuals. In the latest generation of this pedigree, 3 out of 10 individuals are diseased, which are numbered 43, 44 and 48.

**Figure 3:**
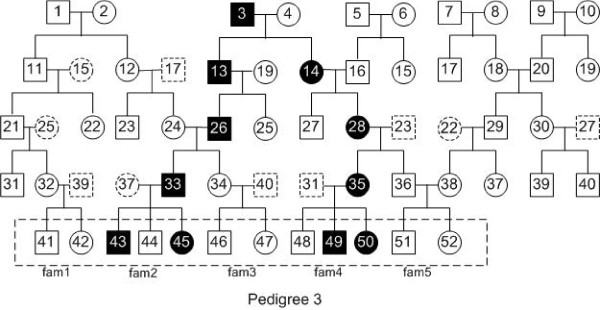
**Pedigree 3: a pedigree with 4 diseased individuals in the input.** There are 5 generations in the pedigree. The filled squares (circles) represent the diseased individuals. In the latest generation of this pedigree, 4 out of 12 individuals are diseased, which are numbered 43, 45, 49 and 50.

**Figure 4:**
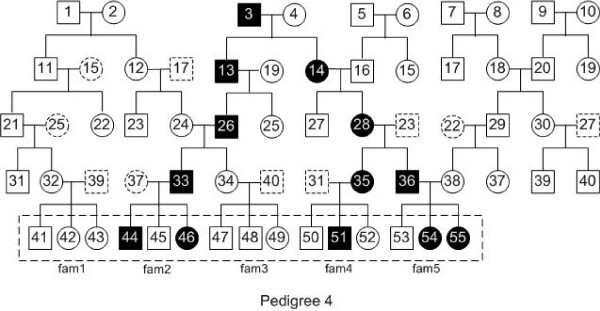
**Pedigree 4: a pedigree with 5 diseased individuals in the input.** There are 5 generations in the pedigree. The filled squares (circles) represent the diseased individuals. In the latest generation of this pedigree, 5 out of 15 individuals are diseased, which are numbered 44, 46, 51, 54 and 55.

To evaluate the performance of our method, we used different pedigrees to evaluate our algorithm. Figures 1, 2, 3 and 4 are pedigrees of 5 generations with 2 to 5 diseased individuals in the dashed rectangle at the bottom. Those individuals in the dashed rectangle at the bottom of each pedigree are the input of our program.

The *correctly detected mutation regions* are the intersection of the regions reported by the computer program and the true mutation region. Here, *precision* is defined as the number of SNPs in the correctly detected mutation regions divided by the total number of SNPs in the regions output by the program. The value of *recall* is defined as the number of SNPs in the correctly detected mutation regions divided by the total number of SNPs in the true mutation region. So, if the value of *recall* is 1, then all the SNPs in the true mutation region have been reported by the program. Similarly, if *precision* is 1, then all the reported SNPs are in the true mutation region.

We performed 200 experiments for each pedigree. Since there are about 110,000 SNP sites on the chromosome, we set *L* = 500. For each region [*rb*,*re*] reported by our algorithm, we define a score as follows: Let *DH* be the number of distinct haplotypes on this region and *LENGTH* = (*re* − *rb* + 1) the length of this region. Then the score of this region is defined as *SCORE* = (2∗*n* − *DH*)∗*LENGTH*, where *n* is the total number of input genotypes in *D*∪*N*. This score can balance the length of the mutation region and the number of distinct haplotype segments on the region. With the longer region and smaller *DH*, the score becomes higher. To illustrate the quality of our program, we report the results when our program reports the region with the highest score and the first three regions with the highest scores, respectively. In fact, our program does not need this score in the computation. The program simply reports all the mutation regions. See the Genotype data error handling in Discussion.

The precision and recall on the experiments are shown in Table 1. Only the genotype of the individuals in the latest generation of Pedigree 1−4 are known in this experiment. Several mutation regions may be detected by our algorithm. In Table 1, the results when our program reports the region with the highest score are shown in the columns under “one region”. The results when our program reports three regions with the highest scores are shown in the columns under “three regions”. The precision and recall are calculated based on the true mutation region, the reported region(s), and the intersection of the reported region(s) and the true mutation region. The precision’ and recall’ are calculated by replacing the true mutation region with shared mutation regions. The column “time” indicates the average time of our program by running 200 experiments on each pedigree.

**Table 1:** Results on input individuals of the latest generation of Pedigrees 1-4

	**One region**				**Three regions**				**Time**
	**Precision**	**Recall**	**Precision’**	**Recall’**	**Precision**	**Recall**	**Precision’**	**Recall’**	
Pedigree 1	29.97%	49.83%	48.79%	54.03%	23.07%	82.71%	40.60%	86.75%	16.80s
Pedigree 2	44.36%	63.09%	58.49%	64.06%	30.66%	93.48%	48.94%	93.18%	17.60s
Pedigree 3	58.54%	80.42%	74.57%	81.07%	34.12%	97.28%	67.73%	97.56%	15.55s
Pedigree 4	75.91%	96.07%	91.27%	96.99%	39.94%	98.07%	86.64%	98.18%	17.89s

From Table 1, we can see that the values of recall are from 82.71% to 98.07% and the values of precision are from 23.07% to 39.94% in the four pedigrees. When the number of diseased individuals is increased, the values of both recall and precision are improved significantly. When there are 4 or 5 diseased individuals, the value of recall is more than 97%. That is, the program can report most of the SNPs in the true mutation region.

In practice, one can often get the genotype data for the individuals of the latest two generations. Thus, we study this case by looking at different input individuals based on the pedigrees in Figures 1, 2, 3 and 4.

**Figure 5:**
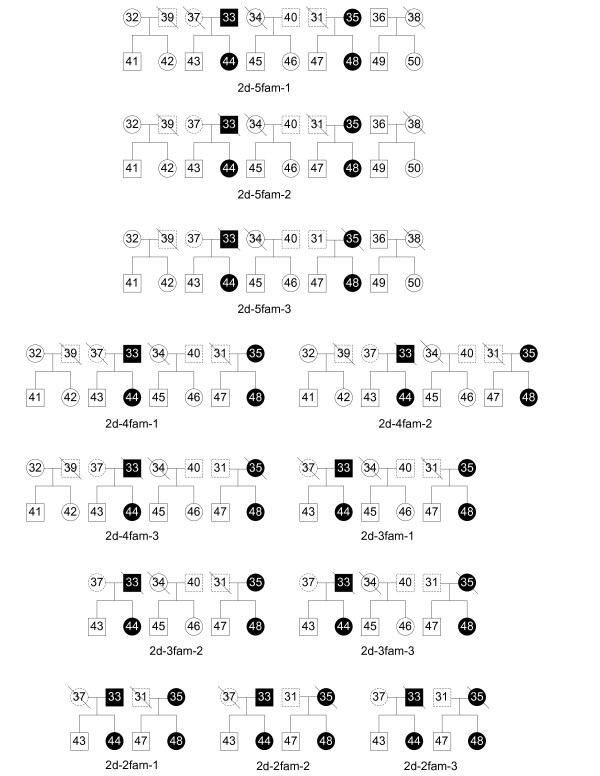
**The different sets of input individuals based on Pedigree 1.** Just the latest two generations are selected in this experiment. Squares and circles with a slash are individuals whose genotype is unknown. From top to bottom, the families in the input decrease.

Now, we study different sets of input individuals in the latest two generations of Pedigree 1. These different sets of input individuals in the latest two generations in the pedigree are given in Figure 5, a square/circle with a slash indicates that such individual is not included as part of the input though the individual is used in generating the simulated data. For the rest of test, we performed 200 experiments for each case and show the average values. Table 2 shows the results for the different sets of input individuals in Figure 5. The individuals in the latest and latest two generations are not distinguished in our algorithm. We just input the genotype for all the individuals without the slash. Again, the setting is similar to that of Table 1. From Table 2, we can see that the values of recall for different inputs are close to 99% except for 2d-3fam-3, 2d-2fam-2 and 2d-2fam-3, where the input contains only 2 or 3 diseased individuals. Comparing Table 1 and Table 2, we can see that more input individuals do help improve the values of precision and recall.

**Table 2:** Results on Figure 5

	**Three regions**				
**Input**	**Precision**	**Recall**	**Precision’**	**Recall’**	**Time**
2d-5fam-1	38.49%	99.85%	78.88%	99.41%	18.62s
2d-5fam-2	39.93%	99.35%	72.21%	99.51%	18.66s
2d-5fam-3	36.04%	95.78%	61.51%	96.61%	19.55s
2d-4fam-1	34.96%	99.77%	70.00%	99.46%	16.10s
2d-4fam-2	26.49%	99.40%	64.78%	99.23%	15.93s
2d-4fam-3	30.48%	94.81%	57.94%	95.87%	17.16s
2d-3fam-1	32.20%	98.75%	66.97%	99.13%	11.73s
2d-3fam-2	32.61%	96.75%	61.95%	97.24%	12.35s
2d-3fam-3	26.58%	89.62%	53.55%	92.88%	13.13s
2d-2fam-1	29.86%	98.97%	65.87%	98.66%	8.21s
2d-2fam-2	26.34%	93.98%	53.01%	93.93%	8.61s
2d-2fam-3	23.10%	88.96%	50.37%	91.97%	9.23s

We also performed similar experiments for Pedigree 2-4 (see Figures 2, 3 and 4). The results are similar to that in Table 2 and are given in Additional file 1.

We also tested the program using pedigrees containing 6 and 7 generations and 2, 3, 4, 5 diseased individuals, respectively, in the latest generation. The four pedigrees containing 6 generations are shown in Additional file 1: Figure S4, Figure S5, Figure S6 and Figure S7 in the Additional file. The four pedigrees containing 7 generations are shown in Additional file 1: Figure S12, Figure S13, Figure S14 and Figure S15 in the Additional file. Again, the input individuals are the individuals in the dashed rectangle at the bottom of the pedigree. The experiment results for 6 generations and 7 generations are shown in Table 3 and Table 4, respectively. The settings of Table 3 and Table 4 are similar to that of Table 1. Table 3 and Table 4 show that the performance of our program for 6 and 7 generations is similar (but slightly worse than) to that for 5 generations.

**Table 3:** Results for the pedigrees containing 6 generations in Additional file 1: Figure S4, Figure S5, Figure S6 and Figure S7 in the Additional file

	**One region**				**Three regions**				**Time**
	**Precision**	**Recall**	**Precision’**	**Recall’**	**Precision**	**Recall**	**Precision’**	**Recall’**	
Pedigree 5	20.28%	36.46%	30.00%	37.94%	18.39%	79.96%	29.15%	80.77%	16.09s
Pedigree 6	26.79%	43.65%	34.74%	43.73%	20.60%	91.63%	31.82%	92.79%	16.40s
Pedigree 7	65.76%	89.97%	80.98%	89.56%	35.47%	97.34%	74.12%	97.11%	16.81s
Pedigree 8	69.21%	91.16%	82.77%	91.34%	36.17%	97.55%	79.53%	97.74%	15.82s

**Table 4:** Results for the pedigrees containing 7 generations in Additional file 1: Figure S12, Figure S13, Figure S14 and Figure S15 in the Additional file

	**One region**				**Three regions**				**Time**
	**Precision**	**Recall**	**Precision’**	**Recall’**	**Precision**	**Recall**	**Precision’**	**Recall’**	
Pedigree 9	25.72%	47.53%	37.12%	46.78%	20.67%	85.42%	34.28%	86.16%	14.66s
Pedigree 10	31.06%	49.51%	44.08%	50.35%	22.10%	90.34%	37.78%	90.59%	13.54s
Pedigree 11	64.78%	88.80%	80.53%	88.45%	34.19%	97.73%	76.43%	97.30%	16.64s
Pedigree 12	71.73%	92.31%	86.48%	92.44%	37.05%	96.31%	82.85%	96.63%	16.15s

Similar to the case of 5 generations, for pedigrees with 6 and 7 generations, we also tested various cases when some individuals of the latest two generations are available as input individuals. The results for 6 and 7 generations are similar to that of 5 generations. The detailed results are given in the Additional file.

### A real case study

**Figure 6:**
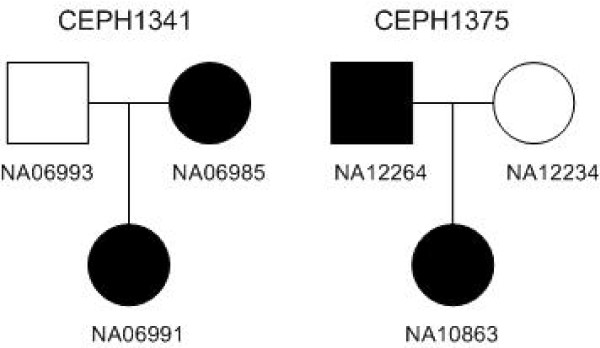
**The pedigree of family CEPH 1341 and CEPH 1375.** The filled individuals are the individuals sharing the segment. We take them as the diseased individuals.

To illustrate the usefulness of our program, we applied our method to a set of real data originally from the phase II HapMap database and was studied in [27]. In [27], the authors studied two CEU (Utah residents with European ancestry from the CEPH collection) families (parent-offspring trios) CEPH 1341 and CEPH 1375 (see Figure 6). They identified a segment (107M to 110M) on chromosome 9 shared by the four individuals NA06991, NA10863, NA06985 and NA12264. For this set of data, there are totally 168,321 SNP sites on the chromosome after the unknown genotypes are eliminated from the database. There are totally 6,519 SNP sites between 107M and 110M on chromosome 9 starting at the 122348-th SNP site and ending at the 128866-th SNP site.

**Figure 7:**
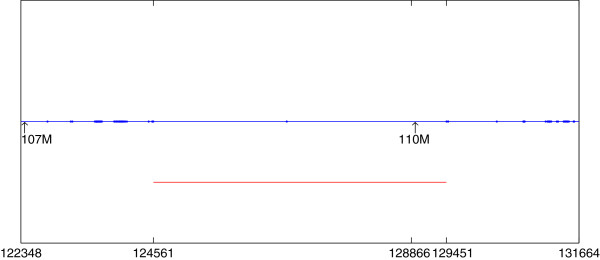
**The region from 107M to 110M on chromosome 9.** There are totally 6,519 SNP sites on this region, as shown by the blue line. The region with the highest score reported by our program contains 4,891 SNP sites, as shown by the red line.

We applied our program with the six individuals in the two families CEPH 1341 and CEPH 1375 as input and set the four individuals NA06991, NA10863, NA06985 and NA12264 as diseased individuals. We set *L* = 500. Our algorithm found several segments shared by the four diseased individuals. The lengths of all the reported segments are approximately 500 SNP sites except the longest ones. All these segments are shown in Table 5. Table 5 shows the starting and ending point of the segments, the number of distinct haplotypes on the segment (DN), and the score for each segment. As there is only one conflicting position 126786 between segments [124561,126785] and [126787,129451], we should consider such a conflicting position as a data error. Therefore, segment [124561,129451] should be the predicted mutation. This segment starts at the 124561-th SNP site and ends at the 129451-th SNP site. The details are shown in Figure 7. We can see that the shared segment found by PLINK in [27] (the blue line) starts at the 122348-th SNP site while the starting position of our reported segment (the red line) is 124561. For the subsegment [122348, 124560] that we did not report, we found 79 conflicting positions in this subsegment containing 2,213 SNP sites. (See the filled dots on the blue line.) However, on the segment [124561, 129451] containing 4,891 SNP sites reported by our program, there is only one conflicting position. This is strong evidence that the subsegment [122348, 124560] is not shared by all the four diseased individuals. We also looked at the segment [129452, 131664] with length of 2,213 SNP sites on the right of our reported segment [124561, 129451], and found 52 conflicting positions among the 2,213 SNP sites. We can see that the subsegments [122348, 124560] and [129452, 131664] on the left and right of our reported segment have approximately the same number of conflicting positions.

**Table 5:** The segments found by our program

**Segment**	**DN**	**Score**
[20935,21543]	8	2436
[41826,42748]	8	3692
[54648,55612]	8	3860
[58385,59106]	8	2888
[59895,60644]	8	3000
[75984,76525]	8	2168
[93972,94699]	9	2184
[97475,98191]	8	2868
[110440,111044]	8	2420
[124561,126785]	8	8900
[126787,129451]	9	7995

## Discussion

### Haplotype inference methods

As discussed before, if the haplotype data for each input individual are known, the problem of finding the true mutation region is straightforward. Currently, there are several population-based phasing methods that can give accurate haplotype segments [28-30]. However, these methods can only phase a small number of SNPs effectively and take an extremely long time to infer the haplotype for the whole chromosome. LRP in [31] can phase more than 1,000 SNPs simultaneously within a reasonable time. However, it is still very slow for phasing 110,000 SNPs of a whole chromosome (as our program does). Moreover, they cannot directly report the true mutation region for a set of input individuals. On the other hand, our program can complete the computation in less than 20 seconds for about 110,000 SNPs with about 10 to 20 individuals.

### Related mutation region detection methods

To our knowledge, all the existing software packages (except PLINK in [27]) need a clearly given pedigree as part of the input. If the pedigree is not known, most of the software packages do not work. Our algorithm deals with the case where the input individuals are closely related but the pedigree is not given.

Merlin is widely used for linkage analysis, where a pedigree is required as part of the input. It works well on SNP data due to the use of sparse trees. However, it can only analyze pedigrees of moderate size. When the family size is big, a large memory space is needed and the computation cannot successfully be completed. As shown in [19], Merlin cannot report the results for some pedigrees, e.g., P14 and P16 in [19], where there are less than 16 input individuals. However, our program can deal with the cases where the number of input individuals is large. The first row in Table 3 of the Additional file shows the results for 20 (not including those without known genotype data) input individuals. We can see that our method can give very high precision and recall in this case (without taking the pedigree as part of the input). Therefore, our algorithm can handle some cases which cannot be handled by Merlin.

The rule-based algorithm in [10] uses a set of heuristics for haplotype inference with a given pedigree. It can give very accurate results when the number of family members is large enough and for each nuclear family the genotype data for both parents are available. However, it does not work well when the genotype data of one of the parents are missing in the nuclear family. If the data for both parents are missing, it does not work. LIden [19] is an extended software package of the algorithm in [10]. It focuses on handling the case where the genotype data for one of the parents in a nuclear family are missing for the entire chromosome. But it still does not work when the genotype data for both parents in a nuclear family are missing or the family pedigree is not given in the input.

PLINK in [27] and Beagle in [30,32] can identify the shared haplotype segment between two individuals based on population-based linkage analysis. But it cannot automatically identify the mutation region taking a set of individuals as the input, which is expected to give more precise prediction. The above real case study has illustrated this.

### Genotype data error handling

The real datasets often contain errors. Handling the genotype data errors is an important issue in practice. For our program, we have a pre-process step to delete all the SNPs containing missing data. That is, if the genotype data for an input individual at an SNP site are missing, we delete this SNP site from the input. Without this step, we cannot get reasonable results for the real case study. When the genotype data contain errors, it is hard to detect and correct them. The errors may affect our program’s results in two ways: (a) an SNP site in the true mutation region may become a conflicting position due to error; and (b) the number of distinct haplotype segments to explain the genotype data is increased. When (b) occurs, our score for the detected mutation regions becomes worse. When (a) occurs once, our program reports two detected regions with the conflicting position in between. See [124561, 126785] and [126787, 129451] in Table 5 of the real case study. When this kind of error occurs many times, our program reports many regions separated by a few SNPs in the middle. When the user looks at the results of our program, it is possible to realize that the few SNPs between two closely located reported regions are due to errors. This is similar to other linkage analysis programs such as Merlin, where each SNP site has a score, and the user decides a region (by ignoring fluctuations) with high scores as the true mutation region.

Our program may work for the situation where the input individuals are from multiple families. Our algorithm tries to find regions shared by all the diseased individuals. Thus, as long as the diseased individuals from multiple families share the same (or similar) haplotype segment on the true mutation region, our program should be able to find such region. Even if the haplotype segments from different families on the true mutation region are slightly different, the program should be able to report several smaller regions with a few missing SNPs in the middle. Again, it is possible for users to figure out the whole true mutation by ignoring the few missing SNPs in the middle.

## Conclusion

We developed a software package for linkage analysis where the input individuals are closely related, but the pedigree is not known. We propose a model using the parsimony principle that can be viewed as an extension of the model first proposed by Dan Gusfield ([20,21]). Our heuristic algorithm simply uses Clark’s inference rule to infer haplotype segments. Experiments show that our program can give very high value (90%-99%) of recall in various cases. This implies that the program can report more than 90% of the true mutation region. The value of precision varies from 29% to 90%. When the precision is 29%, the size of the reported regions is three times that of the true mutation region. This is still very useful for narrowing down the range of the disease gene.

## Availability and requirements

**Project name:** MRD

**Project homepage:**http://www.cs.cityu.edu.hk/~wenjuacui/software/mutationRegion/index.html. The source code is also available.

**Operating system(s):** Platform independent

**Programming language:** Java

**Other requirements:** Java 1.6.0 or higher

**License:** None

**Any restrictions to use by non-academics:** None

## Competing interests

The authors declare that they have no competing interests

## Author’s contributions

LW proposed the topic and ideas for algorithms, WC implemented the program, and both authors devised and developed the method and prepared the manuscript. Both authors read and approved the final manuscript.

## Supplementary Material

Additional file 1 Supplementary Material: This file includes several figures and additional experimental results mentioned in the paper. It contains the different set of input individuals on Pedigree 2-4 in the paper, the pedigrees containing 6 and 7 generations and 2,3,4,5 diseased individuals in the latest generation respectively. The tables show the results on the input of the above figures. Click here for file

## References

[B1] Emahazion T Feuk L Sawyer S Fredman D St Clair D Prince J Brookes A SNP association studies in Alzheimer’s disease highlight problems for complex disease analysis Trends Genet 2001 17 7 407 413 10.1016/S0168-9525(01)02342-3 11418222

[B2] Leykin I Hao K Cheng J Meyer N Pollak M Smith R Wong W Rosenow C Li C Comparative linkage analysis and visualization of high-density oligonucleotide SNP array data BMC Genet 2005 6 7 15713228 10.1186/1471-2156-6-7 PMC551603

[B3] Sellick G Longman C Tolmie J Newbury-Ecob R Geenhalgh L Hughes S Whiteford M Garrett C Houlston R Genomewide linkage searches for Mendelian disease loci can be efficiently conducted using high-density SNP genotyping arrays Nucleic Acids Res 2004 32 20 e164 10.1093/nar/gnh163 15561999 PMC534642

[B4] Lander E Green P Construction of multilocus genetic linkage maps in humans Proc Nat Acad Sci USA 1987 84 8 2363 2367 10.1073/pnas.84.8.2363 3470801 PMC304651

[B5] Kruglyak L Daly M Reeve-Daly M Lander E Parametric and nonparametric linkage analysis: a unified multipoint approach Am J Human Genet 1996 58 6 1347 1363 8651312 PMC1915045

[B6] Gudbjartsson D Jonasson K Frigge M Kong A Allegro, a new computer program for multipoint linkage analysis Nat Genet 2000 25 12 13 10.1038/75514 10802644

[B7] Abecasis G Cherny S Cookson W Cardon L Merlin-rapid analysis of dense genetic maps using sparse gene flow trees Nat Genet 2002 30 97 101 10.1038/ng786 11731797

[B8] Lathrop G Lalouel J Julier C Ott J Strategies for multilocus linkage analysis in humans Proc Nat Acad Sci USA 1984 81 11 3443 3446 10.1073/pnas.81.11.3443 6587361 PMC345524

[B9] Elston R Stewart J A general model for the genetic analysis of pedigree data Human Heredity 1971 21 6 523 542 10.1159/000152448 5149961

[B10] Lin G Wang Z Wang L Lau Y Yang W Identification of linked regions using high-density SNP genotype data in linkage analysis Bioinformatics 2008 24 86 93 10.1093/bioinformatics/btm552 18024969

[B11] Cai Z Sabaa H Wang Y Goebel R Wang Z Xu J Stothard P Lin G Most parsimonious haplotype allele sharing determination BMC Bioinformatics 2009 10 115 10.1186/1471-2105-10-115 19379528 PMC2691739

[B12] Qian D Beckmann L Minimum-recombinant haplotyping in pedigrees Am J Human Genet 2002 70 6 1434 1445 10.1086/340610 11992251 PMC379131

[B13] Tapadar P Ghosh S Majumder P Haplotyping in pedigrees via a genetic algorithm Human Heredity 2000 50 43 56 10.1159/000022890 10545757

[B14] Zhang K Sun F Zhao H HAPLORE: a program for haplotype reconstruction in general pedigrees without recombination Bioinformatics 2005 21 90 103 10.1093/bioinformatics/bth388 15231536

[B15] Doi K Li J Jiang T Minimum recombinant haplotype configuration on tree pedigrees In Proceedings of Workshop on Algorithms in Bioinformatics(WABI) 2003 339 353

[B16] Li J Jiang T Computing the minimum recombinant haplotype configuration from incomplete genotype data on a pedigree by integer linear programming J Comput Biol 2005 12 6 719 739 10.1089/cmb.2005.12.719 16108713

[B17] Li J Jiang T An exact solution for finding minimum recombinant haplotype configurations on pedigrees with missing data by integer linear programming Proceedings of the eighth annual international conference on Resaerch in Computational Molecular Biology(RECOMB) 2004 San Diego, California, USA: ACM 20 29

[B18] Xiao J Liu L Xia L Jiang T Fast elimination of redundant linear equations and reconstruction of recombination-free mendelian inheritance on a pedigree Proceedings of the eighteenth annual ACM-SIAM symposium on Discrete Algorithms 2007 SIAM, New Orleans, Louisiana USA 655 664

[B19] Wang L Wang Z Yang W Linked region detection using high-density SNP genotype data via the minimum recombinant model of pedigree haplotype inference BMC Bioinformatics 2009 10 216 10.1186/1471-2105-10-216 19604391 PMC2723091

[B20] Gusfield D Inference of haplotypes from samples of diploid populations: complexity and algorithms J Comput Biol 2001 8 3 305 323 10.1089/10665270152530863 11535178

[B21] Gusfield D Haplotype inference by pure parsimony Combinatorial Pattern Matching 2003 Morelia, Michocan Mexico: Springer 144 155

[B22] Clark A Inference of haplotypes from PCR-amplified samples of diploid populations Mol Biol Evol 1990 7 2 111 122 2108305 10.1093/oxfordjournals.molbev.a040591

[B23] Wang L Xu Y Haplotype inference by maximum parsimony Bioinformatics 2003 19 14 1773 1780 10.1093/bioinformatics/btg239 14512348

[B24] Brown D Harrower I Integer programming approaches to haplotype inference by pure parsimony IEEE/ACM Trans Comput Biol Bioinformatics 2006 30 2 141 154 10.1109/TCBB.2006.24 17048400

[B25] Broman K Weber J Characterization of human crossover interference Am J Human Genet 2000 66 6 1911 1926 10.1086/302923 10801387 PMC1378063

[B26] Yang W Wang Z Wang L Sham P Huang P Lau Y Predicting the number and sizes of IBD regions among family members and evaluating the family size requirement for linkage studies Eur J Human Genet 2008 16 12 1535 1543 10.1038/ejhg.2008.116 18575462

[B27] Purcell S Neale B Todd-Brown K Thomas L Ferreira M PLINK: a tool set for whole-genome association and population-based linkage analyses Am J Hum Genet 2007 81 559 575 10.1086/519795 17701901 PMC1950838

[B28] Stephens M Smith N Donnelly P A new statistical method for haplotype reconstruction from population data Am J Human Genet 2001 68 4 978 989 10.1086/319501 11254454 PMC1275651

[B29] Scheet P Stephens M A fast and flexible statistical model for large-scale population genotype data: applications to inferring missing genotypes and haplotypic phase Am J Human Genet 2006 78 4 629 644 10.1086/502802 16532393 PMC1424677

[B30] Browning B Browning S A fast, powerful method for detecting identity by descent Am J Human Genet 2011 88 2 173 182 10.1016/j.ajhg.2011.01.010 21310274 PMC3035716

[B31] Kong A Masson G Frigge M Gylfason A Zusmanovich P Thorleifsson G Olason P Ingason A Steinberg S Rafnar T Detection of sharing by descent, long-range phasing and haplotype imputation Nature Genet 2008 40 9 1068 1075 10.1038/ng.216 19165921 PMC4540081

[B32] Browning S Browning B High-resolution detection of identity by descent in unrelated individuals Am J Human Genet 2010 86 4 526 539 10.1016/j.ajhg.2010.02.021 20303063 PMC2850444

