# Supplementary material for: Identifying mutation regions for closely related individuals without a known pedigree
Source: BMC Bioinformatics. 2012 Jun 25;13:146. doi: 10.1186/1471-2105-13-146 (PMC3507658; doi:10.1186/1471-2105-13-146)
Supplement: Additional file 1 — Supplementary Material: This file includes several figures and additional experimental results mentioned in the paper. It contains the different set of input individuals on Pedigree 2-4 in the paper, the pedigrees containing 6 and 7 generations and 2,3,4,5 diseased individuals in the latest generation respectively. The tables show the results on the input of the above figures. [file 1471-2105-13-146-S1.pdf]

Supplementary Material for "Identifying Mutation Regions for Closely Related Individuals without a Known Pedigree"

1 The experiments for pedigrees containing 5 generations

We study different sets of input individuals in the latest two generations of Pedigree 2-4 in the paper, where there are 5 generations in those pedigrees. Those different sets of input individuals in the latest two generations in the pedigree are given in Figures 1-3. We just input the genotype for the individuals without the slash. The results when our program reports three regions with the highest scores are shown in Table 1-3. The precision and recall are calculated based on the true mutation region, the reported region(s) and the intersection of the reported region(s) and the true mutation region. The precision' and recall' are calculated by replacing the true mutation region with shared mutation regions. The column "time" indicates the average time of our program by running 200 experiments on each set of input. We can see that the values of recall are very close to 100% in all the cases. The value of precision is getting better when the number of diseased individuals increases. Our program can give the output in less than 20 seconds.

2 The experiments for pedigrees containing 6 generations

We also do experiments on the pedigrees containing 6 generations. Here we consider Pedigree 5-8 as shown in Figure 4-7. The results are shown in Table 3 in the paper.

We study different sets of input individuals in the latest two generations of Pedigree 5-8, where there are 6 generations in those pedigrees. Those different sets of input individuals in the latest two generations in the pedigree

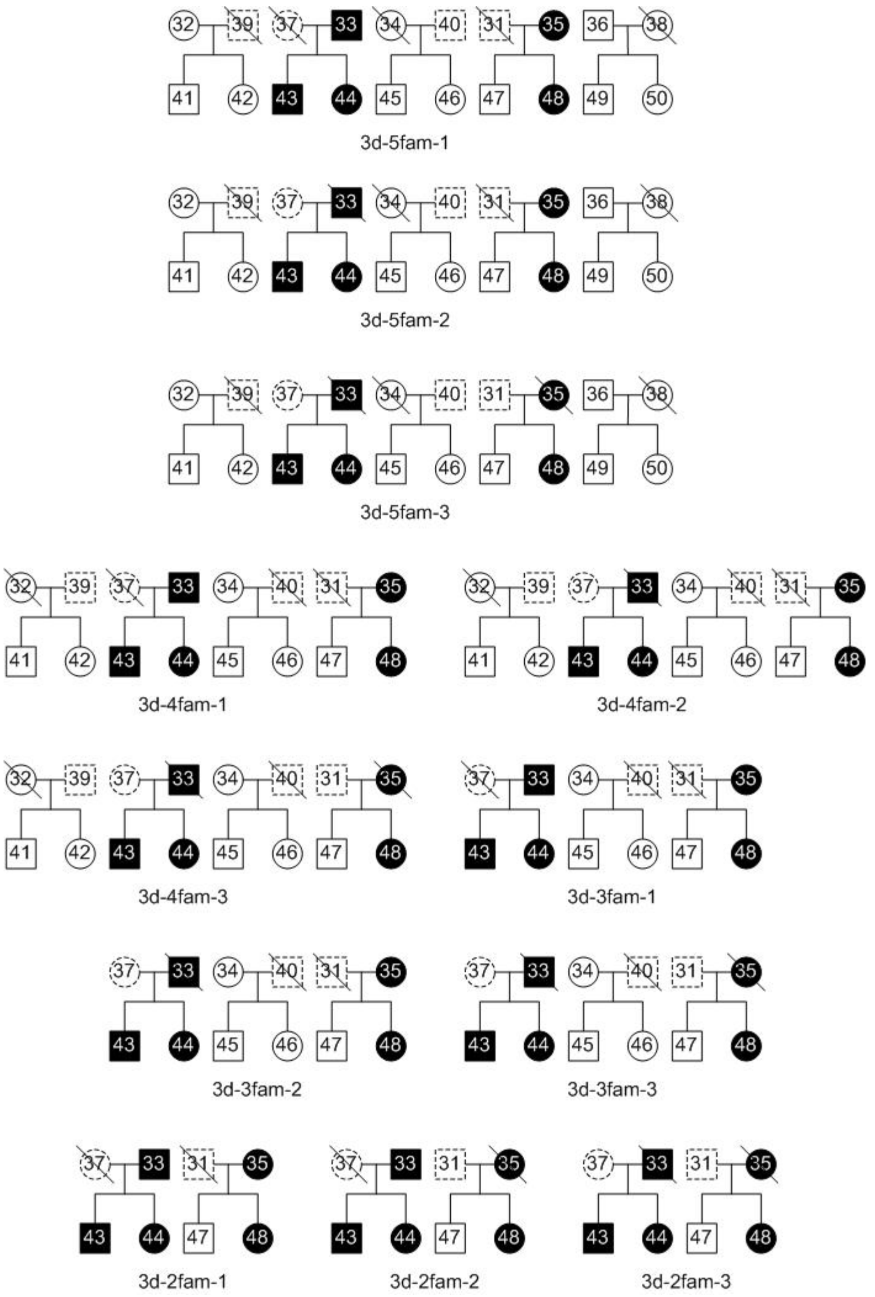

Figure 1: The different sets of input individuals based on Pedigree 2 in the paper.

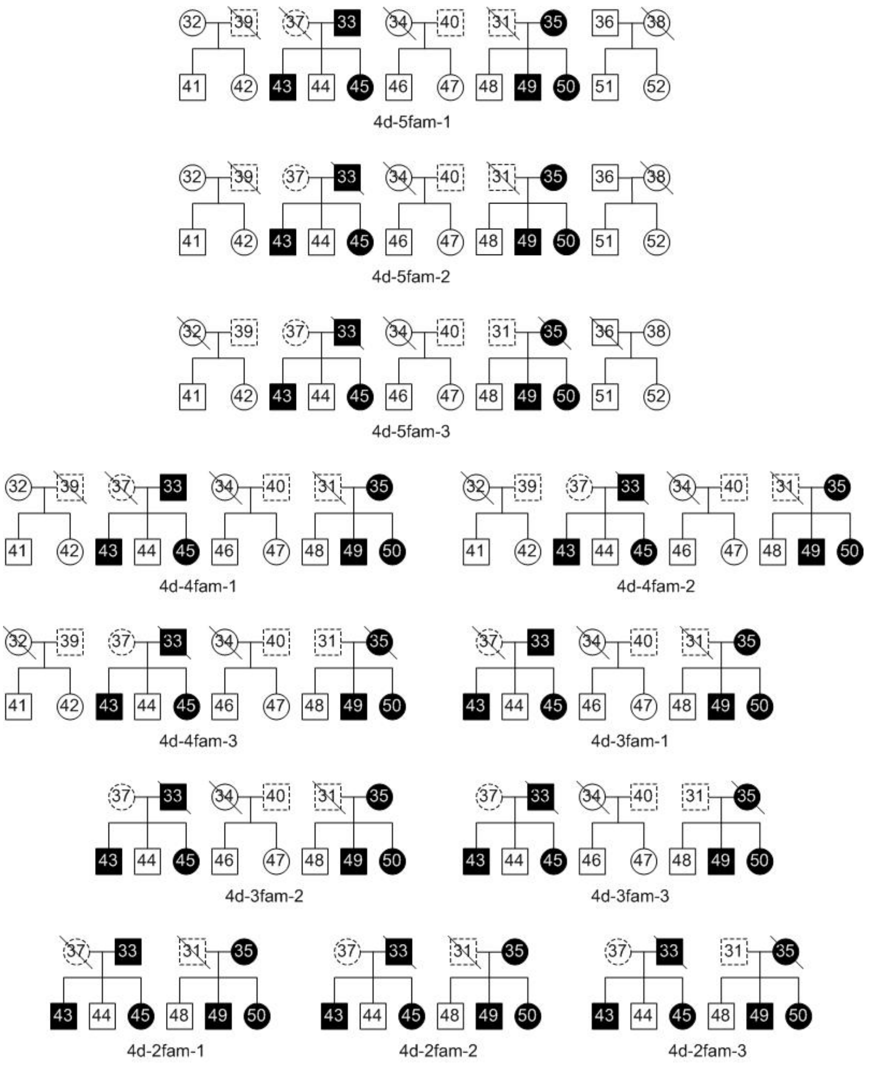

Figure 2: The different sets of input individuals based on Pedigree 3 in the paper.

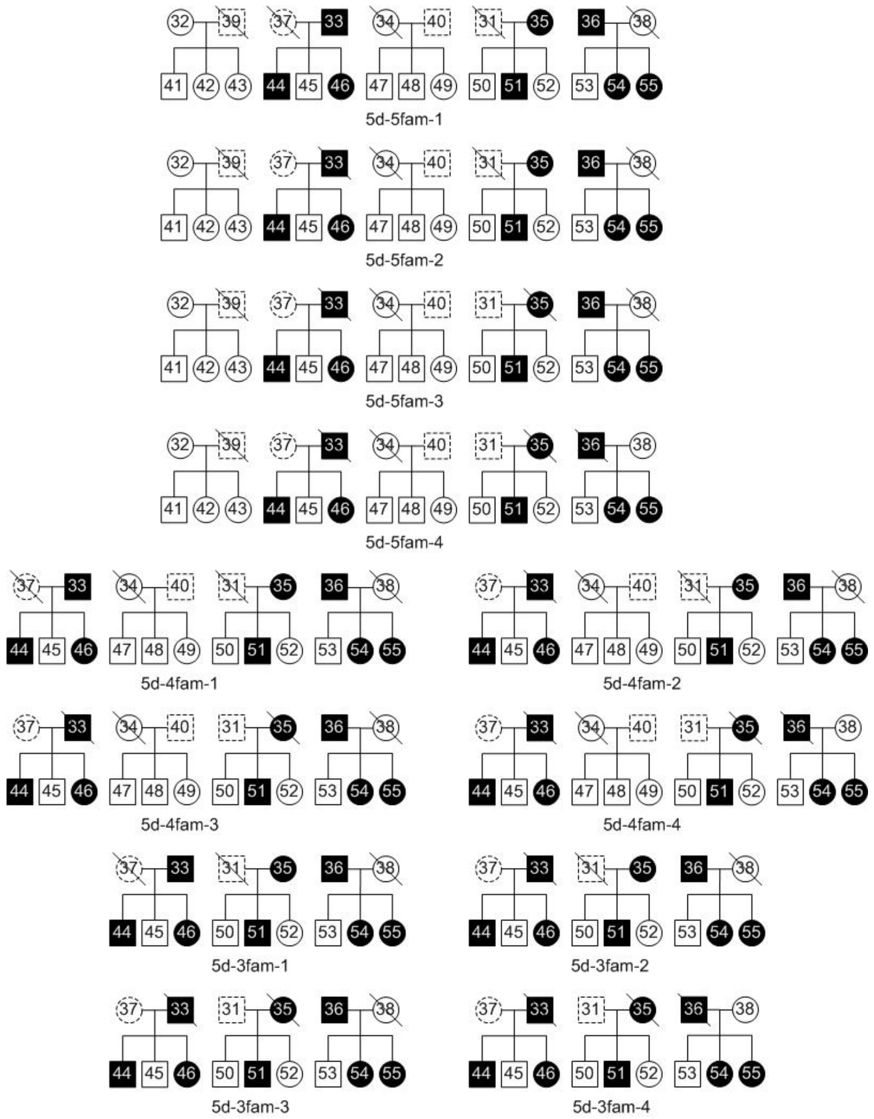

Figure 3: The different sets of input individuals based on Pedigree 4 in the paper.

three regions					
input	precision	recall	precision'	recall'	time
3d-5fam-1	40.32%	99.40%	79.83%	99.04%	15.53s
3d-5fam-2	40.69%	98.90%	74.71%	98.54%	15.88s
3d-5fam-3	39.87%	97.94%	65.29%	97.73%	16.63s
3d-4fam-1	38.01%	99.37%	78.80%	99.27%	12.93s
3d-4fam-2	38.40%	98.86%	68.07%	98.63%	13.23s
3d-4fam-3	35.57%	96.41%	59.62%	95.75%	14.22s
3d-3fam-1	36.54%	99.42%	76.77%	99.26%	9.57s
3d-3fam-2	35.98%	98.93%	66.28%	98.66%	9.85s
3d-3fam-3	31.89%	95.50%	56.29%	95.06%	12.29s
3d-2fam-1	35.20%	98.00%	72.23%	97.99%	6.55s
3d-2fam-2	30.81%	95.50%	57.69%	95.53%	7.01s
3d-2fam-3	27.50%	93.50%	54.67%	94.32%	6.93s

Table 1: Results on Figure 1

three regions					
input	precision	recall	precision'	recall'	time
4d-5fam-1	40.03%	96.13%	87.44%	96.53%	17.13s
4d-5fam-2	40.32%	96.76%	85.13%	97.11%	16.41s
4d-5fam-3	39.07%	97.15%	80.54%	97.41%	18.95s
4d-4fam-1	38.55%	96.10%	85.85%	96.70%	14.53s
4d-4fam-2	39.15%	96.64%	82.04%	97.08%	14.00s
4d-4fam-3	38.21%	96.81%	78.82%	97.68%	14.41s
4d-3fam-1	37.35%	96.20%	83.81%	96.49%	11.94s
4d-3fam-2	38.07%	96.29%	80.54%	96.58%	12.49s
4d-3fam-3	36.57%	96.83%	75.52%	97.58%	11.72s
4d-2fam-1	35.46%	95.40%	83.78%	96.37%	8.23s
4d-2fam-2	35.99%	95.81%	79.76%	96.76%	10.70s
4d-2fam-3	34.83%	96.35%	74.53%	97.45%	10.48s

Table 2: Results on Figure 2

three regions					
input	precision	recall	precision'	recall'	time
5d-5fam-1	40.53%	98.60%	91.35%	99.26%	18.90s
5d-5fam-2	40.65%	98.61%	90.79%	99.12%	18.63s
5d-5fam-3	40.50%	98.58%	90.55%	99.14%	19.18s
5d-5fam-4	41.07%	98.53%	88.67%	99.08%	19.67s
5d-4fam-1	39.29%	98.76%	91.02%	99.40%	15.02s
5d-4fam-2	39.80%	98.77%	90.43%	99.22%	15.09s
5d-4fam-3	39.89%	98.74%	90.56%	99.21%	15.05s
5d-4fam-4	40.35%	98.69%	88.76%	99.26%	15.34s
5d-3fam-1	37.95%	99.01%	92.26%	99.42%	12.02s
5d-3fam-2	38.63%	99.00%	92.25%	99.18%	16.29s
5d-3fam-3	38.56%	98.97%	92.16%	98.97%	11.52s
5d-3fam-4	38.86%	98.91%	89.84%	99.19%	11.39s

Table 3: Results on Figure 3

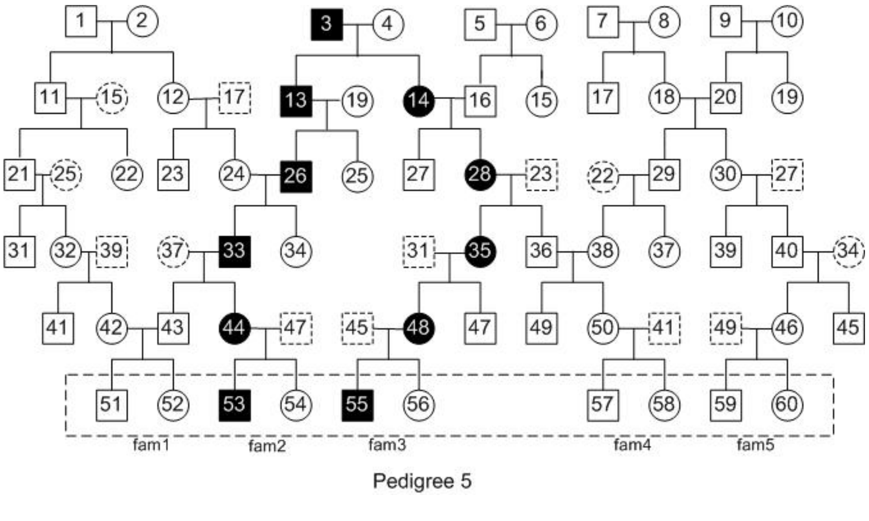

Figure 4: Pedigree 5: a pedigree containing 6 generations with 2 diseased individuals in the input.

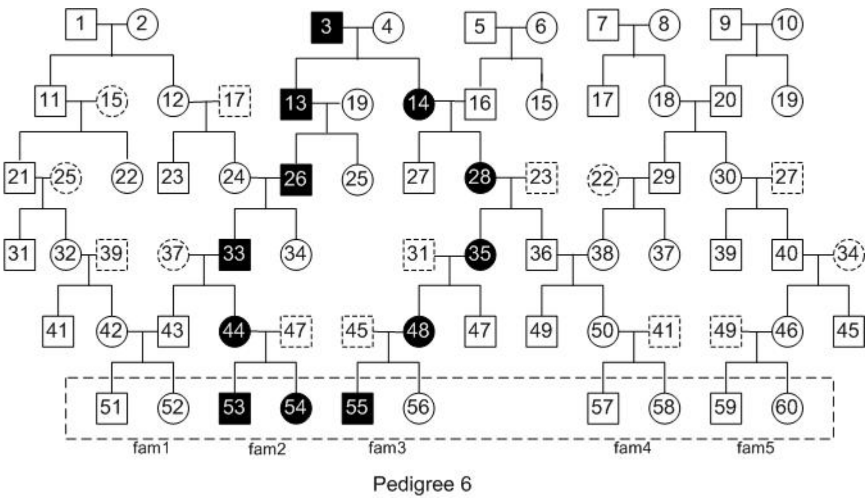

Figure 5: Pedigree 6: a pedigree containing 6 generations with 3 diseased individuals in the input.

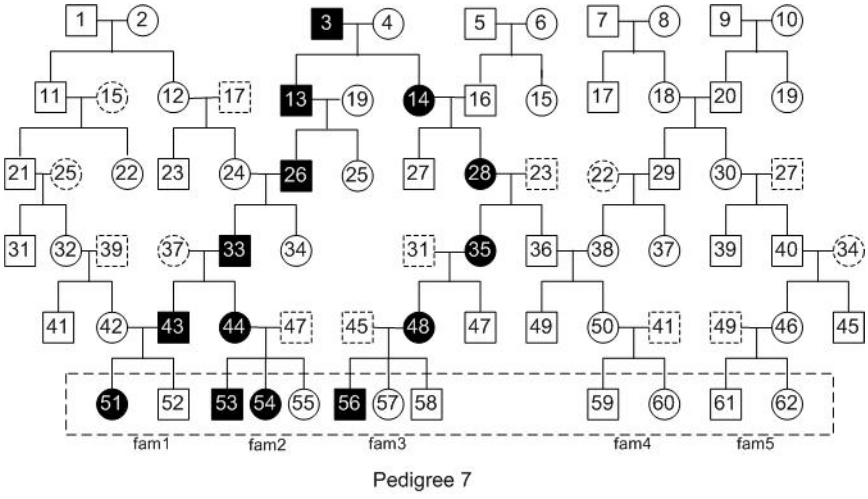

Figure 6: Pedigree 7: a pedigree containing 6 generations with 4 diseased individuals in the input.

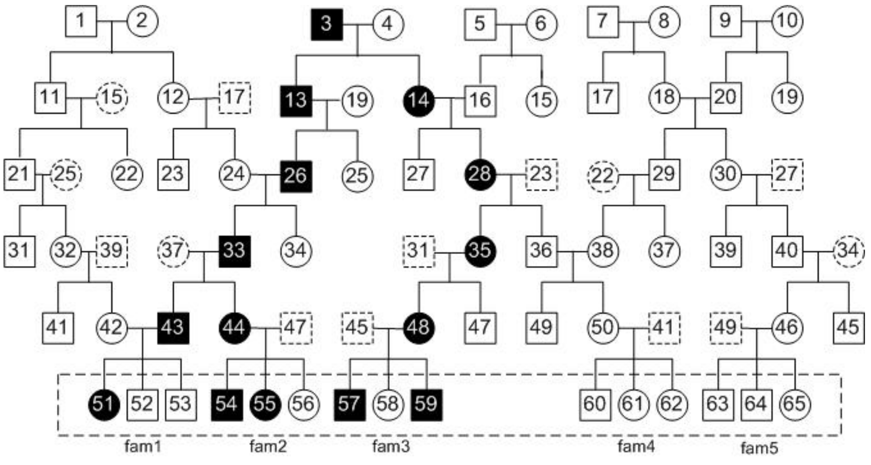

Figure 7: Pedigree 8: a pedigree containing 6 generations with 5 diseased individuals in the input.

are given in Figures 8-11. The results are shown in Table 4-7. The setting of the tables are similar to Table 1-3. We can see that the values of recall are more than 90% in most cases. The value of precision is getting better when the number of diseased individuals increases. The behavior is similar to that of 5 generations.

3 The experiments for pedigrees containing 7 generations

The pedigrees shown in Figure 12-15 contain 7 generations and 2, 3, 4, 5 diseased individuals in the latest generation. Only the individuals in the latest generation are the input individuals. The experiment results are shown in Table 4 in the paper.

Figure 16-19 show the different sets of input individuals in the latest two generations of Pedigree 9-12. The results are shown in Table 8-11. The setting of the tables are similar to Table 1-3. The performance of our program for 7 generations is similar to that for 5 and 6 generations but slightly worse than them. We do 200 experiments for each set of input individuals mentioned above.

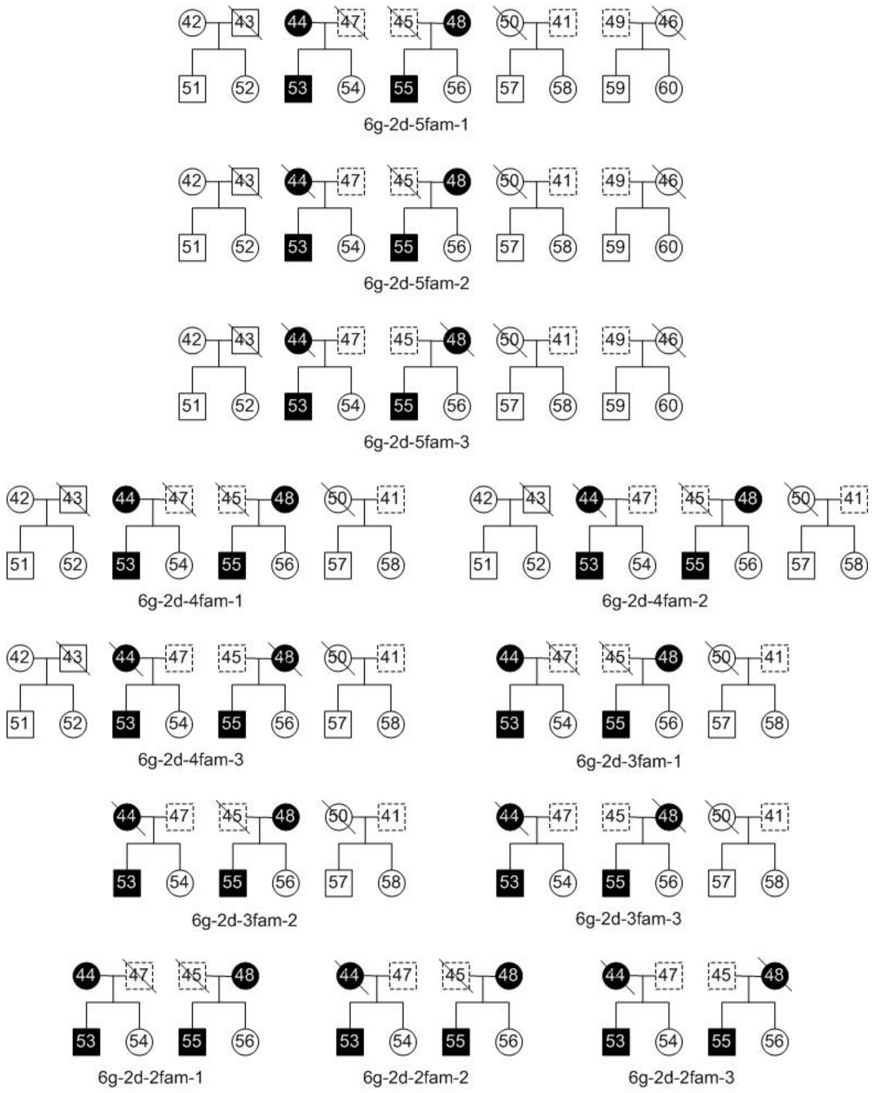

Figure 8: The different sets of input individuals based on Pedigree 5

three regions					
input	precision	recall	precision'	recall'	time
6g-2d-5fam-1	37.00%	98.39%	72.38%	98.34%	16.37s
6g-2d-5fam-2	34.50%	98.89%	61.48%	98.25%	16.74s
6g-2d-5fam-3	34.10%	97.90%	53.47%	97.43%	17.81s
6g-2d-4fam-1	34.94%	98.45%	69.38%	98.43%	13.40s
6g-2d-4fam-2	31.43%	97.93%	56.54%	97.75%	14.11s
6g-2d-4fam-3	30.53%	97.07%	49.21%	96.76%	14.89s
6g-2d-3fam-1	31.63%	97.97%	65.74%	98.38%	10.80s
6g-2d-3fam-2	25.56%	96.36%	47.10%	96.59%	11.59s
6g-2d-3fam-3	22.61%	89.48%	41.66%	89.74%	12.45s
6g-2d-2fam-1	28.89%	96.97%	61.65%	97.47%	7.65s
6g-2d-2fam-2	19.24%	89.47%	37.10%	90.78%	8.19s
6g-2d-2fam-3	17.05%	78.91%	34.01%	80.63%	8.91s

Table 4: Results on Figure 8

three regions					
input	precision	recall	precision'	recall'	time
6g-3d-5fam-1	34.96%	97.80%	70.18%	97.40%	16.90s
6g-3d-5fam-2	35.41%	98.31%	65.75%	98.17%	17.21s
6g-3d-5fam-3	34.01%	97.40%	54.38%	97.12%	17.77s
6g-3d-4fam-1	33.25%	98.38%	66.27%	98.00%	14.41s
6g-3d-4fam-2	31.21%	96.87%	58.36%	96.53%	15.13s
6g-3d-4fam-3	29.29%	95.90%	49.09%	95.46%	14.79s
6g-3d-3fam-1	30.71%	97.46%	62.53%	97.36%	11.34s
6g-3d-3fam-2	26.38%	95.42%	50.73%	95.05%	11.95s
6g-3d-3fam-3	22.97%	90.96%	41.44%	91.49%	12.49s
6g-3d-2fam-1	29.11%	95.96%	60.18%	96.28%	7.82s
6g-3d-2fam-2	22.68%	91.87%	45.59%	92.51%	8.05s
6g-3d-2fam-3	18.10%	82.46%	35.73%	83.58%	9.46s

Table 5: Results on Figure 9

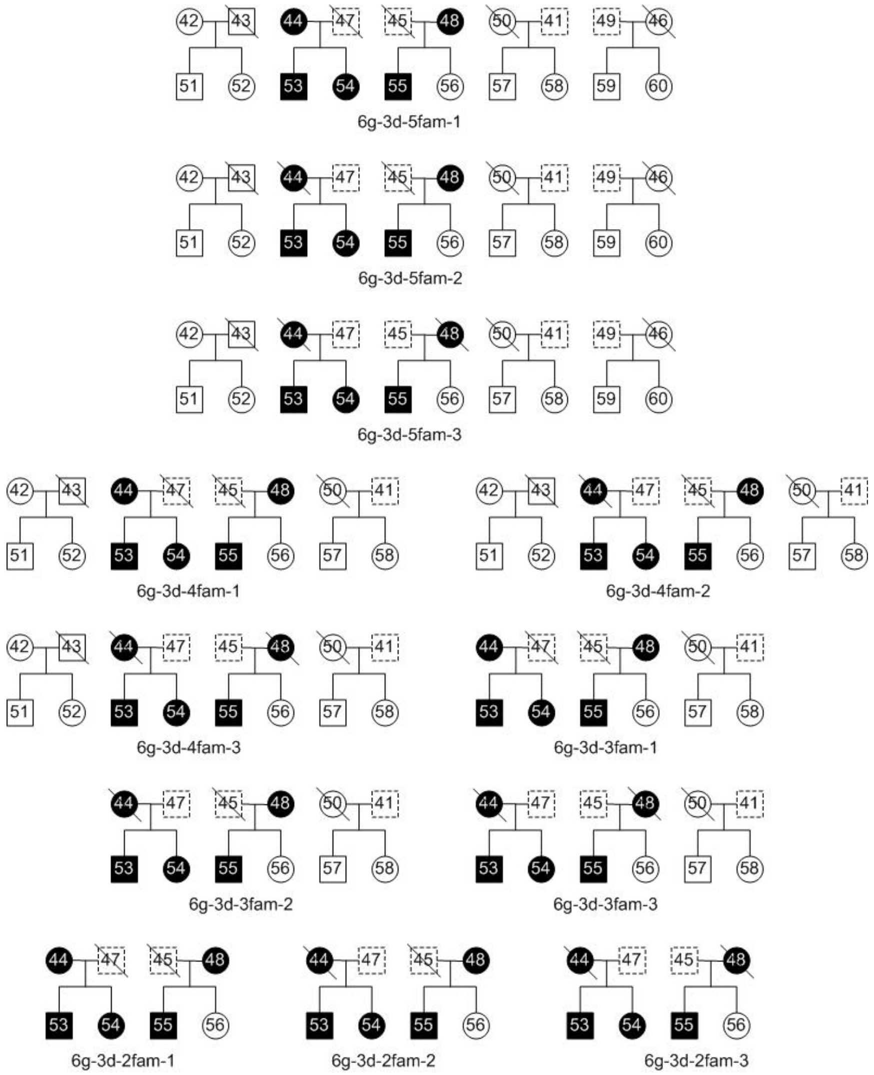

Figure 9: The different sets of input individuals based on Pedigree 6

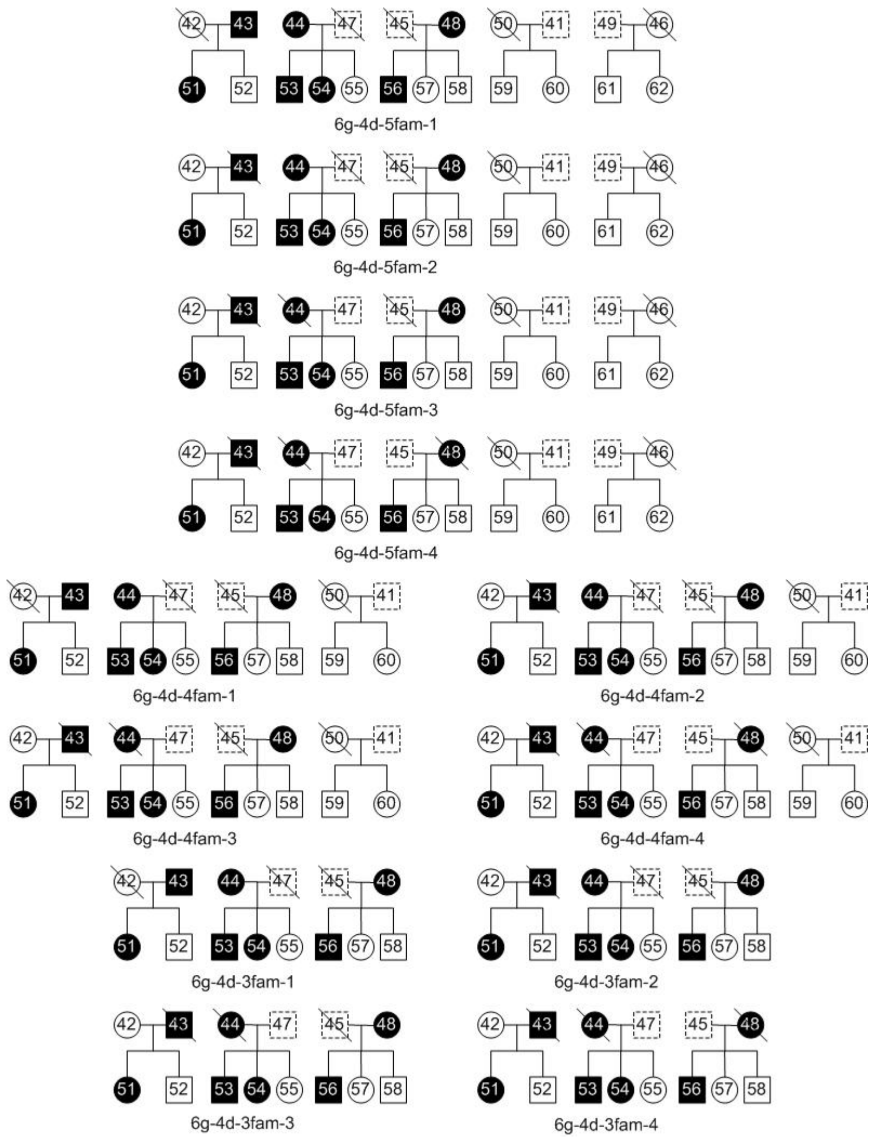

Figure 10: The different sets of input individuals based on Pedigree 7

three regions					
input	precision	recall	precision'	recall'	time
6g-4d-5fam-1	38.73%	97.12%	90.16%	97.10%	17.85s
6g-4d-5fam-2	38.88%	97.08%	90.04%	97.16%	18.13s
6g-4d-5fam-3	39.04%	97.27%	89.05%	97.20%	17.88s
6g-4d-5fam-4	38.52%	97.75%	84.16%	97.71%	17.98s
6g-4d-4fam-1	37.65%	97.72%	90.58%	97.42%	14.56s
6g-4d-4fam-2	37.67%	97.71%	90.14%	97.49%	15.09s
6g-4d-4fam-3	37.74%	97.89%	88.74%	97.83%	15.16s
6g-4d-4fam-4	36.79%	98.41%	82.41%	98.34%	18.64s
6g-4d-3fam-1	36.71%	97.92%	90.30%	97.63%	14.72s
6g-4d-3fam-2	36.79%	97.91%	89.41%	97.69%	13.43s
6g-4d-3fam-3	36.97%	98.03%	87.61%	98.01%	10.22s
6g-4d-3fam-4	35.59%	98.54%	81.54%	98.51%	11.00s

Table 6: Results on Figure 10

three regions					
input	precision	recall	precision'	recall'	time
6g-5d-5fam-1	38.85%	96.34%	89.56%	96.58%	17.41s
6g-5d-5fam-2	39.09%	96.34%	88.91%	96.58%	16.83s
6g-5d-5fam-3	39.15%	96.35%	87.72%	96.35%	18.60s
6g-5d-5fam-4	38.92%	96.99%	87.28%	97.08%	18.39s
6g-5d-4fam-1	37.65%	96.67%	89.56%	97.08%	15.31s
6g-5d-4fam-2	37.89%	96.67%	89.11%	97.05%	14.23s
6g-5d-4fam-3	37.74%	96.66%	87.69%	96.85%	14.16s
6g-5d-4fam-4	37.80%	97.16%	86.67%	97.08%	14.60s
6g-5d-3fam-1	36.71%	96.91%	89.42%	97.08%	10.68s
6g-5d-3fam-2	37.14%	96.91%	89.05%	97.08%	10.40s
6g-5d-3fam-3	37.06%	97.39%	88.04%	97.35%	10.17s
6g-5d-3fam-4	37.11%	97.89%	86.40%	97.58%	10.33s

Table 7: Results on Figure 11

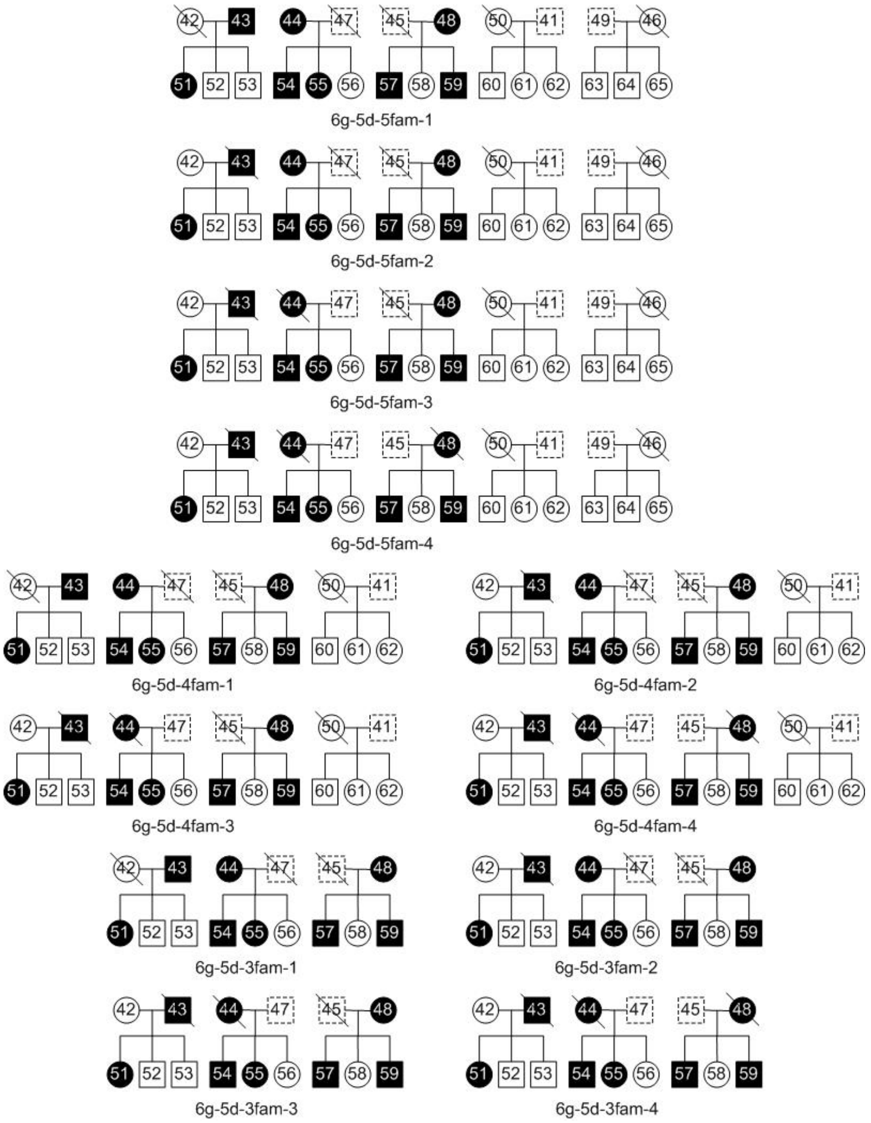

Figure 11: The different sets of input individuals based on Pedigree 8

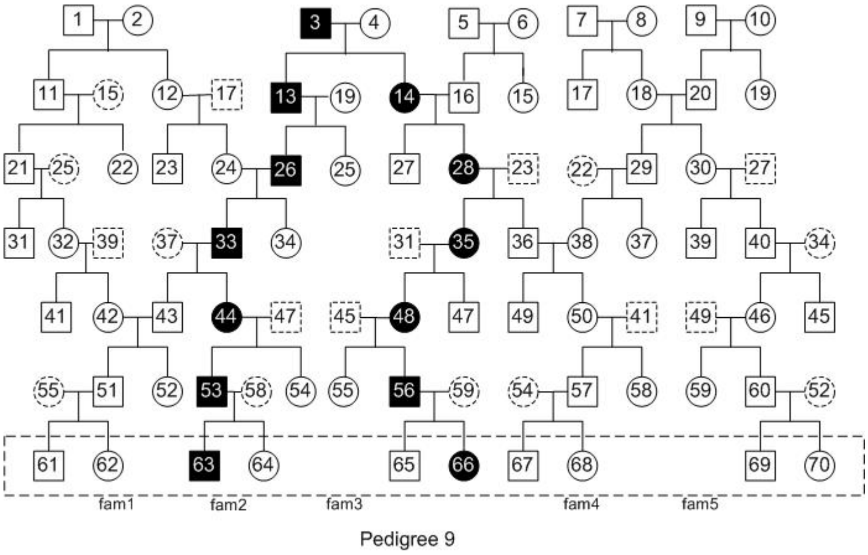

Figure 12: Pedigree 9: a pedigree containing 7 generations with 2 diseased individuals in the input.

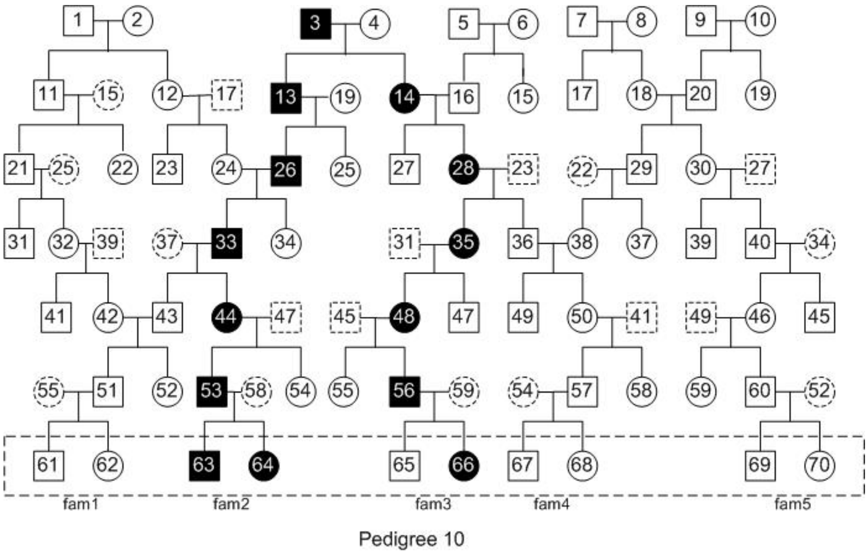

Figure 13: Pedigree 10: a pedigree containing 7 generations with 3 diseased individuals in the input.

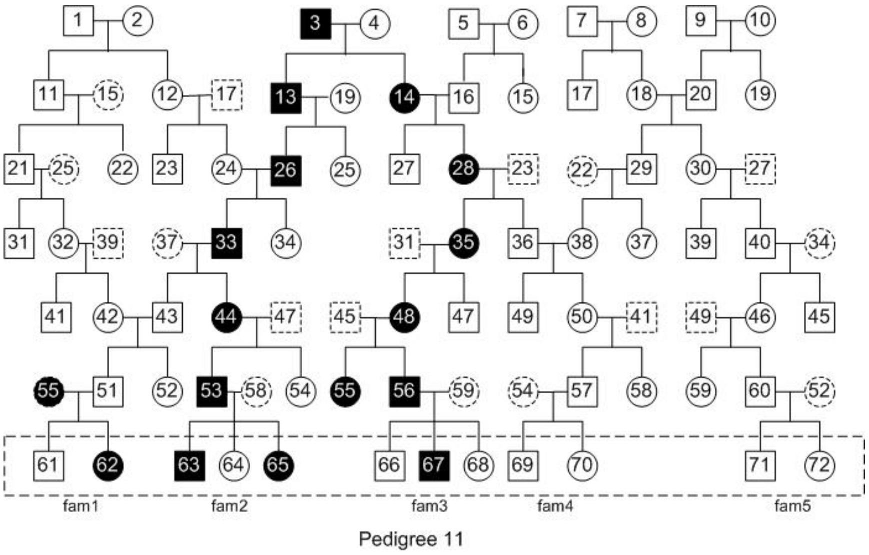

Figure 14: Pedigree 11: a pedigree containing 7 generations with 4 diseased individuals in the input.

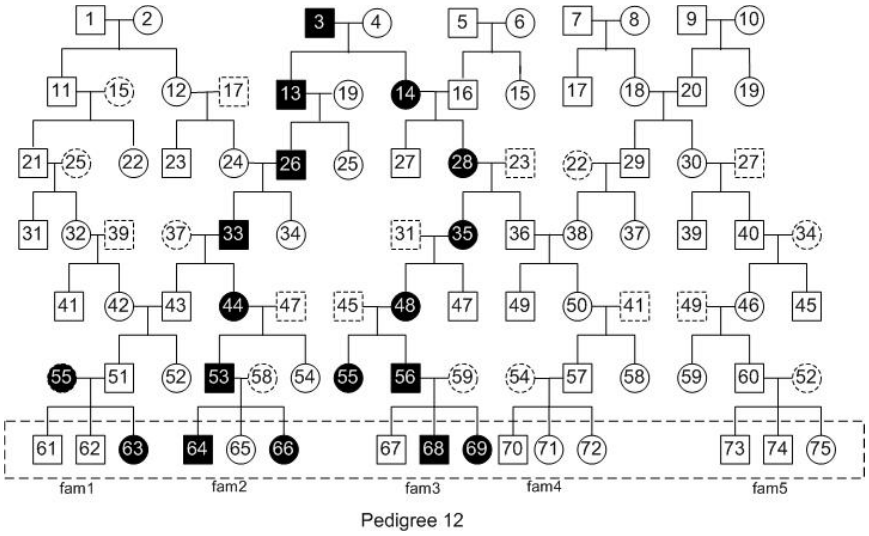

Figure 15: Pedigree 12: a pedigree containing 7 generations with 5 diseased individuals in the input.

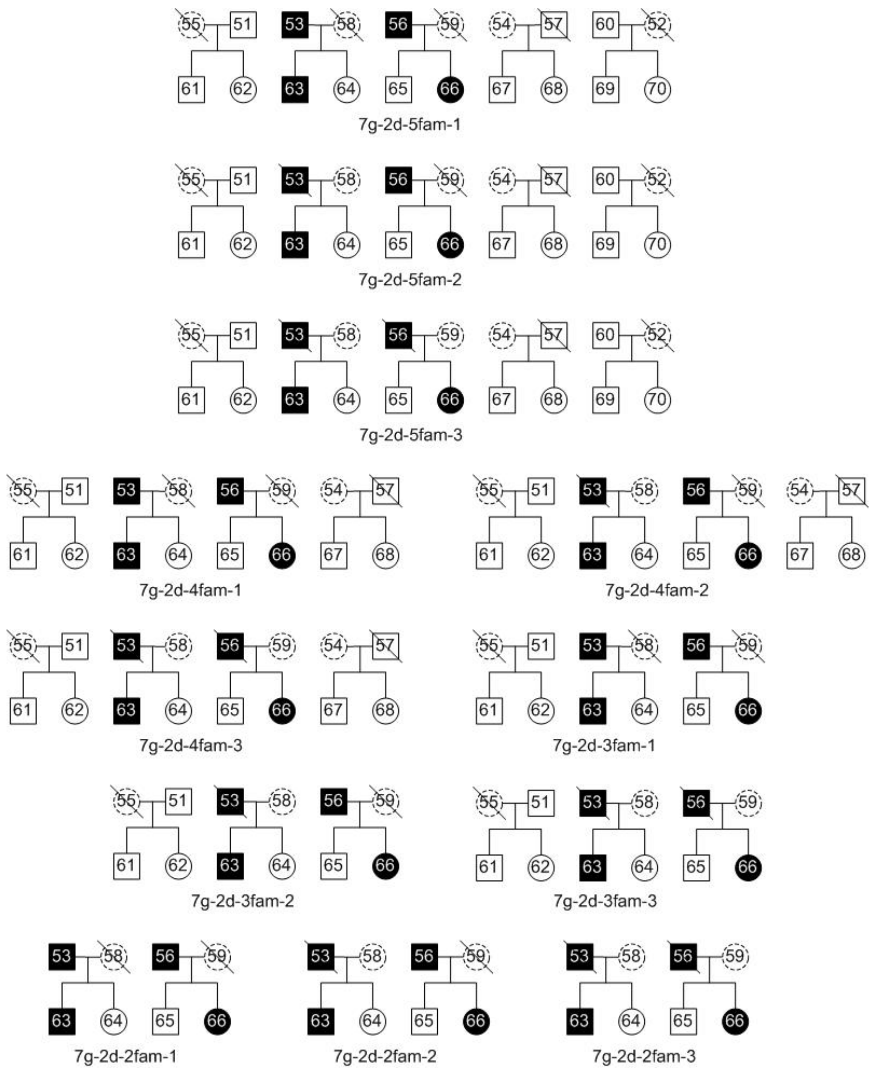

Figure 16: The different sets of input individuals based on Pedigree 9

three regions					
input	precision	recall	precision'	recall'	time
7g-2d-5fam-1	33.63%	97.16%	74.83%	96.79%	13.48s
7g-2d-5fam-2	32.39%	96.68%	64.95%	96.80%	13.76s
7g-2d-5fam-3	31.03%	97.85%	55.08%	97.41%	14.80s
7g-2d-4fam-1	31.47%	97.19%	69.49%	96.87%	11.10s
7g-2d-4fam-2	29.37%	96.69%	58.55%	96.87%	11.51s
7g-2d-4fam-3	27.82%	95.87%	50.80%	96.32%	11.98s
7g-2d-3fam-1	27.01%	95.43%	58.11%	95.29%	8.71s
7g-2d-3fam-2	24.41%	93.06%	51.23%	94.57%	9.08s
7g-2d-3fam-3	22.25%	91.59%	43.30%	93.78%	9.59s
7g-2d-2fam-1	22.11%	91.95%	48.75%	92.04%	5.98s
7g-2d-2fam-2	19.03%	87.93%	46.30%	92.14%	6.38s
7g-2d-2fam-3	16.41%	86.95%	37.80%	87.33%	6.73s

Table 8: Results on Figure 16

three regions					
input	precision	recall	precision'	recall'	time
7g-3d-5fam-1	37.30%	98.47%	77.44%	98.57%	13.21s
7g-3d-5fam-2	35.29%	98.56%	72.88%	98.67%	13.55s
7g-3d-5fam-3	33.84%	98.09%	61.93%	97.89%	14.06s
7g-3d-4fam-1	34.55%	98.03%	72.40%	98.27%	10.93s
7g-3d-4fam-2	31.95%	98.62%	66.60%	98.76%	11.05s
7g-3d-4fam-3	30.78%	97.64%	54.46%	97.34%	11.54s
7g-3d-3fam-1	29.64%	97.27%	57.48%	97.76%	8.63s
7g-3d-3fam-2	26.74%	95.85%	57.71%	96.57%	8.60s
7g-3d-3fam-3	24.45%	92.34%	45.69%	92.68%	9.48s
7g-3d-2fam-1	26.03%	95.87%	50.98%	95.24%	5.95s
7g-3d-2fam-2	23.08%	91.95%	55.19%	94.11%	5.99s
7g-3d-2fam-3	18.92%	87.00%	42.25%	89.19%	6.45s

Table 9: Results on Figure 17

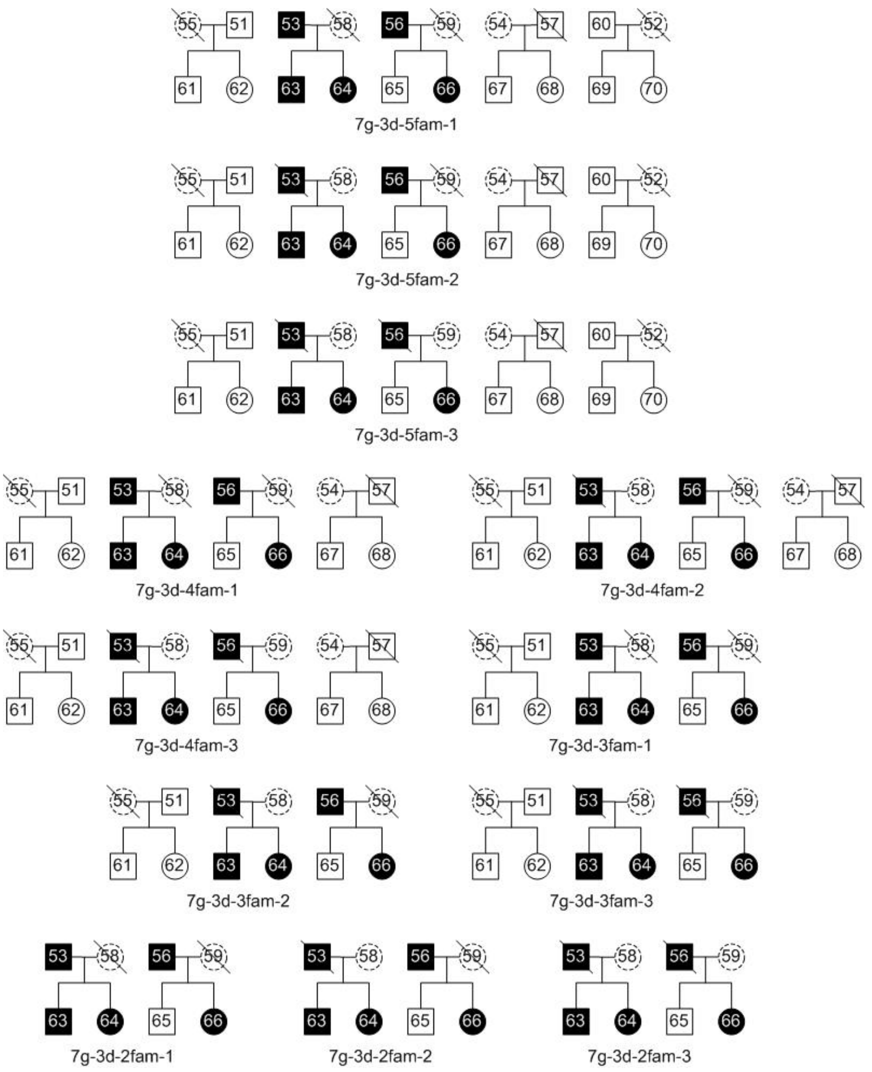

Figure 17: The different sets of input individuals based on Pedigree 10

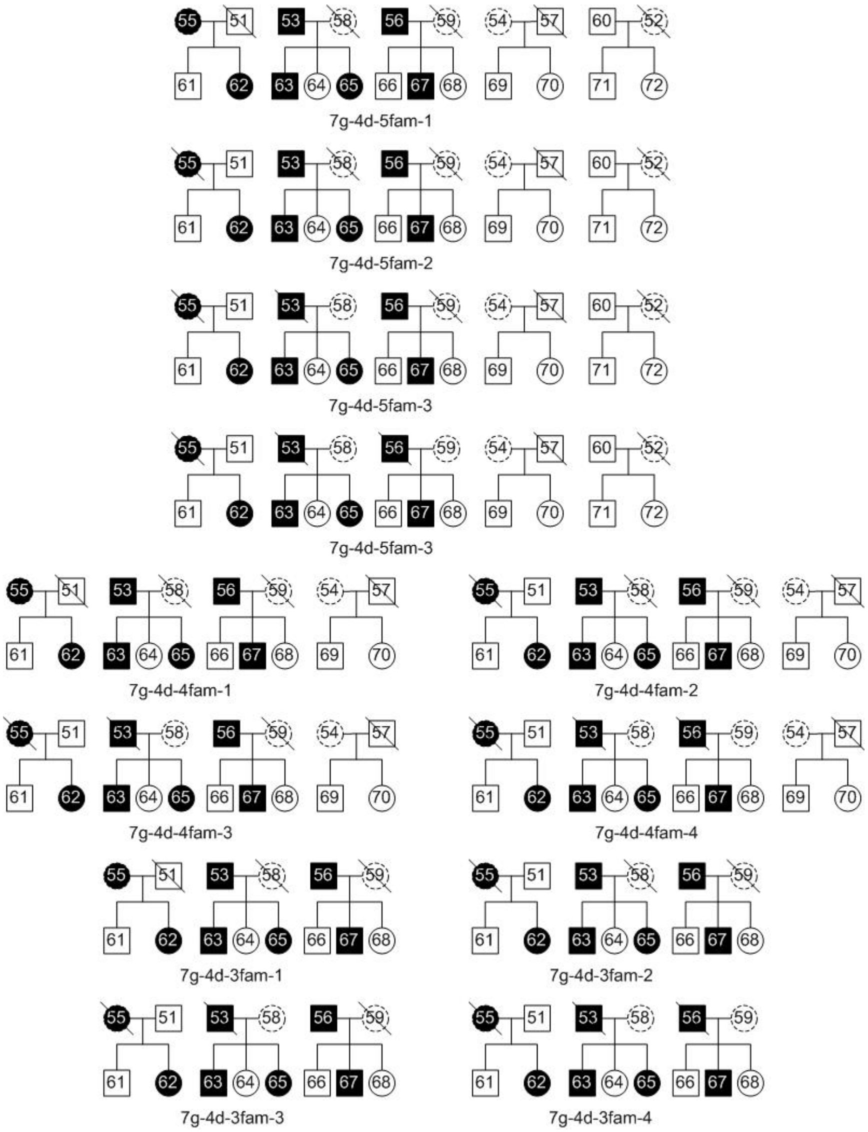

Figure 18: The different sets of input individuals based on Pedigree 11

three regions					
input	precision	recall	precision’	recall’	time
7g-4d-5fam-1	36.77%	97.16%	87.51%	97.21%	14.59s
7g-4d-5fam-2	37.20%	97.15%	88.43%	97.25%	14.49s
7g-4d-5fam-3	37.25%	97.70%	86.93%	97.37%	14.52s
7g-4d-5fam-4	36.71%	97.70%	85.25%	97.30%	14.56s
7g-4d-4fam-1	35.92%	97.18%	87.53%	97.28%	11.77s
7g-4d-4fam-2	36.34%	97.18%	88.58%	97.18%	11.78s
7g-4d-4fam-3	36.45%	97.73%	86.63%	97.41%	11.79s
7g-4d-4fam-4	35.35%	97.73%	83.31%	97.40%	11.81s
7g-4d-3fam-1	34.50%	97.28%	85.66%	97.12%	9.47s
7g-4d-3fam-2	34.64%	97.28%	86.29%	97.28%	9.43s
7g-4d-3fam-3	34.61%	97.78%	85.34%	97.43%	9.68s
7g-4d-3fam-4	34.19%	97.78%	81.05%	97.32%	9.49s

Table 10: Results on Figure 18

three regions					
input	precision	recall	precision’	recall’	time
7g-5d-5fam-1	38.52%	96.22%	91.02%	96.57%	17.96s
7g-5d-5fam-2	38.73%	96.32%	91.20%	96.66%	17.32s
7g-5d-5fam-3	38.61%	96.36%	89.45%	96.70%	17.30s
7g-5d-5fam-4	38.62%	96.36%	88.32%	96.46%	17.37s
7g-5d-4fam-1	37.39%	96.51%	90.85%	96.92%	13.65s
7g-5d-4fam-2	37.89%	96.62%	91.22%	96.88%	13.71s
7g-5d-4fam-3	37.47%	96.60%	88.87%	96.92%	13.81s
7g-5d-4fam-4	37.47%	96.60%	87.45%	96.59%	13.87s
7g-5d-3fam-1	35.60%	96.68%	89.62%	96.99%	10.24s
7g-5d-3fam-2	36.31%	96.79%	90.33%	96.98%	10.59s
7g-5d-3fam-3	36.05%	96.29%	89.24%	96.79%	10.73s
7g-5d-3fam-4	36.54%	96.79%	87.51%	96.94%	10.47s

Table 11: Results on Figure 19

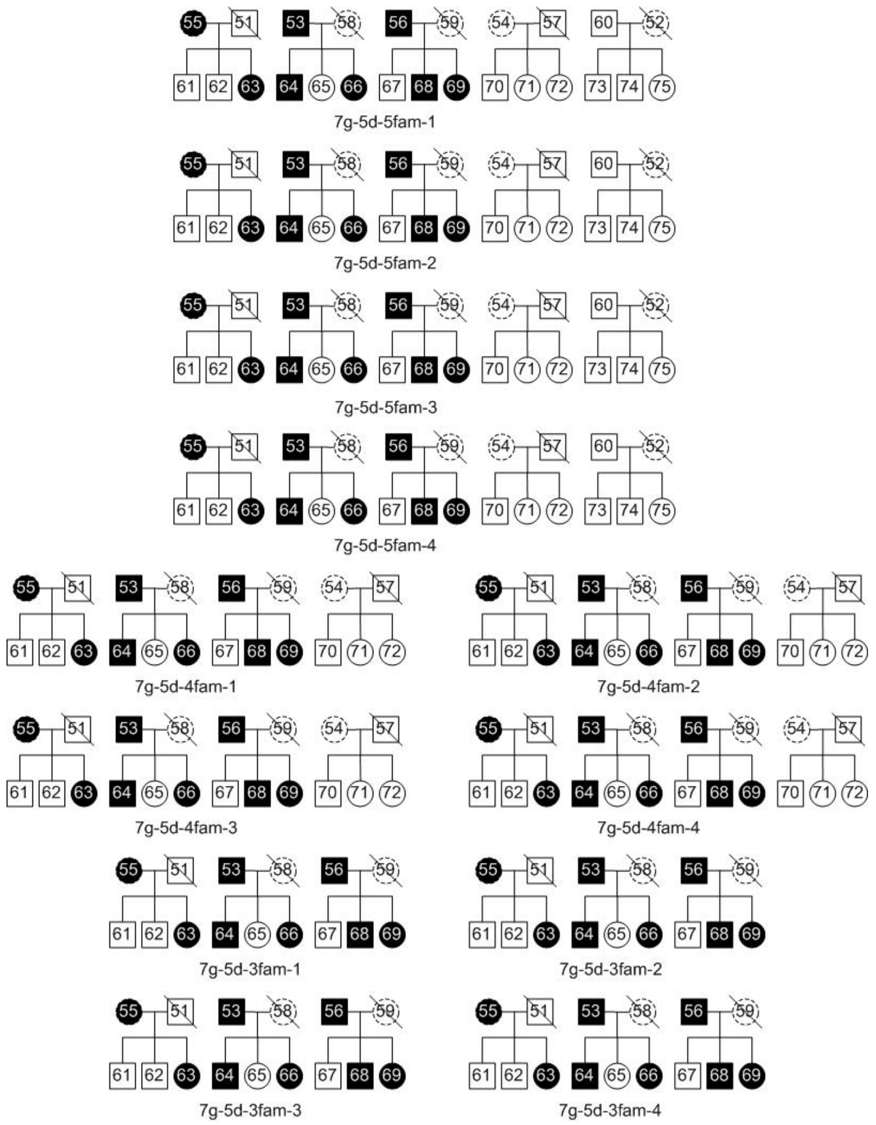

Figure 19: The different sets of input individuals based on Pedigree 12
